# Self-generated chemotaxis of mixed cell populations

**DOI:** 10.1073/pnas.2504064122

**Published:** 2025-08-21

**Authors:** Mehmet Can Uçar, Zane Alsberga, Jonna Alanko, Michael Sixt, Edouard Hannezo

**Affiliations:** ^a^Institute of Science and Technology Austria, Klosterneuburg 3400, Austria

**Keywords:** cell migration, chemotaxis, Keller–Segel model, traveling waves, nonreciprocity

## Abstract

Cell and tissue movement in development, cancer invasion, and immune response relies on chemical or mechanical guidance cues. In many systems, this behavior is locally directed by self-generated signaling gradients rather than long-range, prepatterned cues. However, how heterogeneous mixtures of cells interact nonreciprocally and navigate through self-generated gradients remains largely unexplored. Here, we introduce a theoretical framework for the self-organized chemotaxis of heterogeneous cell populations. We find that the relative chemotactic sensitivities of different cell populations control their long-time coupling and comigration dynamics, with boundary conditions such as external cell and attractant reservoirs substantially influencing the migration patterns. Our model predicts an optimal parameter regime that enables robust and colocalized migration. We test our theoretical predictions with in vitro experiments demonstrating the comigration of distinct immune cell populations, and quantitatively reproduce observed migration patterns under wild-type and perturbed conditions. Interestingly, immune cell comigration occurs close to the predicted optimal regime. Finally, we incorporate mechanical interactions into our framework, revealing a nontrivial interplay between chemotactic and mechanical nonreciprocity in driving collective migration. Together, our findings suggest that self-generated chemotaxis is a robust strategy for the navigation of mixed cell populations.

## Significance

The coordinated movement of cells drives essential processes like tissue development, immune response, and cancer invasion. Rather than relying on prepatterned cues, many cell populations navigate using self-generated chemical or mechanical signals. While much is known about migration of a single cell type, how mixed cell populations coordinate their movement to reach specific targets remains unclear. Here, we combine theoretical modeling with experiments on mixtures of distinct immune cell types to show that self-generated chemotaxis enables efficient long-range migration while optimizing physical interactions. Our findings suggest that this mechanism provides a robust and adaptable strategy for heterogeneous cell populations to navigate complex environments.

Directional cell and tissue movement controls many key processes in development and disease such as morphogenesis, immune response, and cancer invasion. While it is commonly assumed that this collective motion is steered by prepatterned chemical or mechanical cues, only limited evidence has been gathered to support such long-range guidance in vivo (1). In contrast, recent findings are unveiling the prominence of local, self-generated cues across a wide array of in vitro and in vivo settings (2–8), prompting a shift toward exploring controlled vs. self-organized guidance as alternative mechanisms of collective cellular dynamics (9).

Although recent experimental and computational studies have established self-generated chemotactic gradients as a robust mechanism for long-range guidance (10), most work has focused either on homogeneous cell populations or on phenotypic heterogeneity within single-species systems, such as bacterial migration (11–14). However, directional cell migration often involves coordination among distinct cell types, such as during processes like cell sorting (15), morphogenesis (16–20), wound healing (21), cancer invasion and growth (22, 23), and immune cell migration (8). In the latter context, we recently showed that dendritic cells (DCs) can both generate and follow a self-generated chemoattractant gradient, whereas T cells appear to only respond to it (8), raising the question of how such distinct immune cell types coordinate their migration. Overall, the ubiquity of multicomponent cell communication raises the fundamental question on the role of self-organized guidance cues in navigating mixed cell populations toward specific targets.

From a theoretical standpoint, recent research has focused on the dynamics of chemotactic invasion with cell growth (24–26), and on clarifying the existence or stability of traveling concentration fronts of single populations (12, 25–29). Some recent studies have also started to explore variability in cells’ chemotactic responses (11, 12, 14). However, for a comprehensive understanding of multicomponent navigation strategies via self-generated chemotaxis, it is essential to consider mixed cell types with distinct roles, such as chemotactic cell populations acting as sinks or sources in patterning (30).

Here, we propose a theoretical framework to explore the self-generated chemotaxis of mixed cell populations, concentrating on a sink-sensor system where two cell types are (asymmetrically) coupled through a diffusible chemoattractant. We find a rich spectrum of qualitatively distinct comigration patterns, governed by a few key dimensionless parameters, such as relative chemotactic strength of different populations.

In particular, our phase diagram predicts that robust and efficient comigration (defined as both cell populations migrating at the same speed and being spatially colocalized) occurs in a specific region of the parameter space, characterized by their relative chemotactic strength being larger than, but close to, unity. We also explore the role of interactions with external cell and attractant “reservoirs” in the system, taking two limits of initially fixed and renewing attractant levels, and find that these give rise to qualitatively different collective dynamics. Interestingly, we find that cell populations migrate as traveling waves only in the absence of an external attractant reservoir, where self-generated gradients remain sharp enough to facilitate robust front propagation. We furthermore theoretically predict the propagation velocity and density profiles of coupled cell populations, and quantitatively test these predictions using different controlled in vitro assays with mixtures of distinct immune cell types consisting of DCs and T cells. Our framework offers a simple mechanism by which self-generated gradients can drive sustained interactions and optimal migration of heterogeneous cell populations.

## Results

### Continuum Model of Chemotactic Cell Mixtures.

**Fig. 1.**
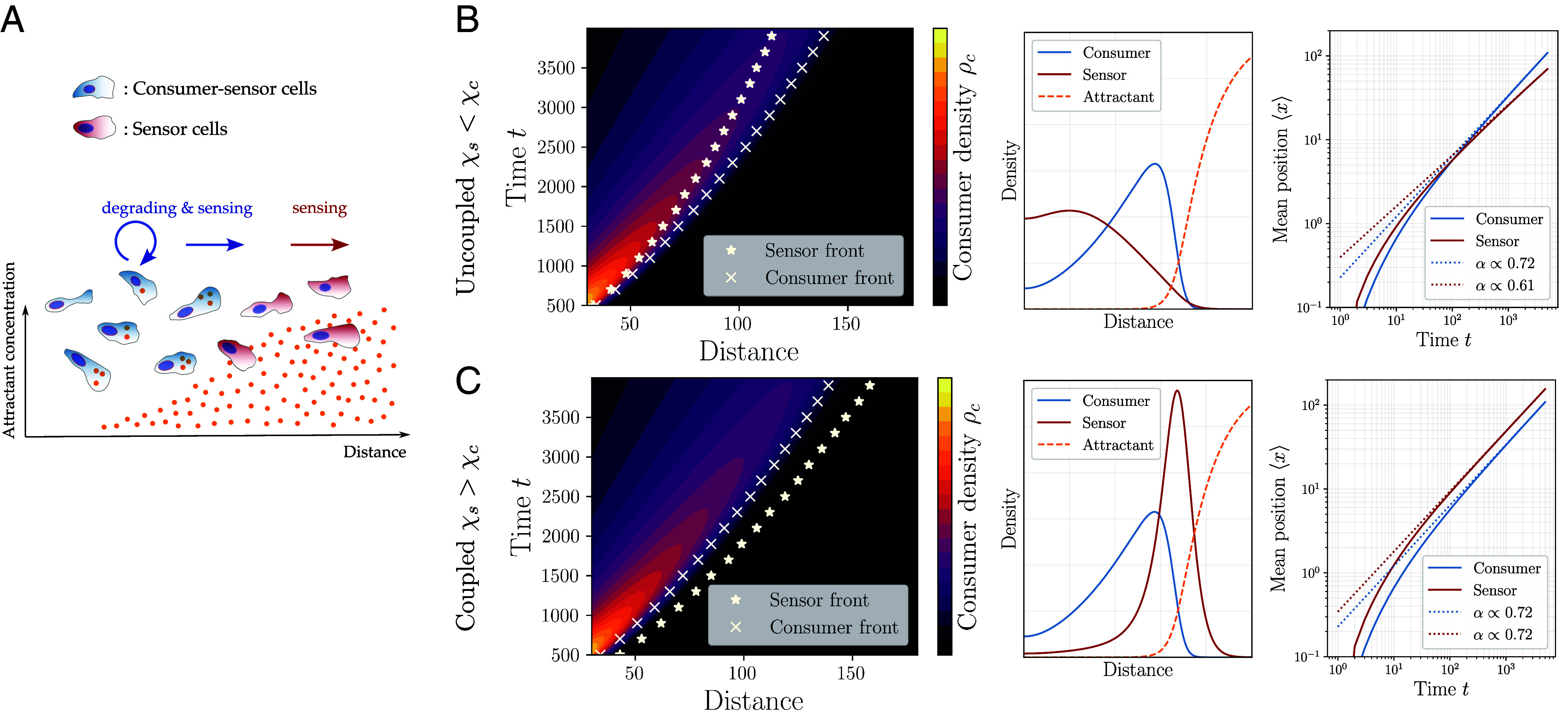
Coupled chemotaxis of mixed cell populations interacting through a locally modified attractant concentration. (*A*) Schematics of the theoretical model. Chemoattractant concentration (illustrated at the *Bottom*) is dynamically shaped by *consumer-sensor cells* (blue) that can locally internalize the ligand, and preferentially migrate toward higher chemoattractant concentration. *Sensor cells* (red) only read off and respond to the local attractant gradient, but cannot modulate it. (*B*) If the rescaled chemotactic coefficient ${\tilde{\chi }}_{s}$ of the sensor cells is smaller than the coefficient ${\tilde{\chi }}_{c}$ of the consumers, the sensor cells cannot follow the migratory front of the consumer cells, leading to the breakdown of their effective coupling through the attractant. Front positions (crosses and star symbols) are based on the half-maximum of the cell density profiles. (*Left*) Kymographs show that the sensor-cell front (star symbols) falls behind the consumer-cell front (crosses) for large times. (*Middle*) Density profile of the sensor cells (red) does not exhibit a well-defined peak and is confined to the back of the consumer cells (blue). (*Right*) The long-time behavior of the mean position as given by the relation $\left\langle x\right\rangle \propto t^{\alpha }$ shows that the asymptotic exponent of sensor cells attains a smaller value than that of the consumer cells, i.e. $\alpha_{s}<\alpha_{c}$. (*C*) If the rescaled chemotactic coefficient of the sensor cells is larger than that of the consumers, i.e. for ${\tilde{\chi }}_{s}>{\tilde{\chi }}_{c}$, sensor cells migrate robustly ahead of the consumer cell front over long distances. (*Left*) Kymographs show that the sensor front is coupled to the consumer-cell front over large times. (*Middle*) Spatial profiles of cell populations exhibit coupled propagation of well-defined density peaks. (*Right*) Long-time scaling of the mean positions shows that the scaling exponents *α* of consumer and sensor cells are superdiffusive (i.e. α>0.5) attaining similar values $\alpha_{s}≃\alpha_{c}≃0.7$, which indicates their asymptotic coupling.

To explore the migration dynamics of heterogeneous cell populations, we turn to a continuum modeling approach adequate to describe cell and chemoattractant dynamics in a coarse-grained framework, extending the seminal work of Keller and Segel (31). We focus on a minimal model for two distinct populations of chemotactic cells types: i) Consumer-sensor cells that can actively modulate as well as sense gradients of the chemoattractant, which we will call consumer cells in short, and ii) sensor cells that only sense and respond to it, with densities denoted by $\rho_{c}$ and $\rho_{s}$, respectively; see Fig. 1*A* for an illustration. Effectively, this means that the first cell population is able to perform self-generated chemotaxis on its own, while the second population needs to “surf” on the gradient generated by the first one. The density evolutions of the cells are determined by an advection–diffusion equation (see *SI Appendix* for details):

[1] $$\begin{array}{c} \partial_{t}\rho_{i}=D_{i}\nabla^{2}\rho_{i}-\nabla \cdot \left(\rho_{i}v_{i}\right)\;, \end{array}$$

with the chemotactic drift velocity

[2] $$\begin{array}{c} v_{i}\equiv \chi_{i}\frac{\nabla a}{a}\;, \end{array}$$

where $D_{i}$ and $\chi_{i}$ denote the diffusion and the chemotactic coefficients of the cell type i=s,c, and *a* denotes the chemoattractant concentration. This logarithmic sensing represents the simplest form of a Weber–Fechner type response, in the absence of more detailed information on the receptor kinetics of cells (14, 29, 32). However, we also directly infer the specific form of chemotactic response from experimental cell density profiles, providing empirical support for this choice (see below). This type of relative sensing could also align with dimensional estimates from the Berg–Purcell limit (33), which suggest that large immune cells like DCs can reliably detect shallow gradients of fast-diffusing chemoattractants even at low concentrations. We provide a brief discussion and model predictions for alternative response models, including absolute and bounded logarithmic forms, in *SI Appendix*, sections S1 and S2.

The time evolution of the attractant *a* is determined by its diffusion and internalization by the consumer cell population. In the absence of an external reservoir, attractant density then follows:

[3] $$\begin{array}{c} \partial_{t}a=D_{a}\nabla^{2}a-m\rho_{c}a\;, \end{array}$$

where $D_{a}$ represents the diffusion coefficient of the chemoattractant, and *m* is the uptake rate of chemoattractant molecules by the consumer cells. The consumer cell population actively shapes the attractant profile, thereby controlling the chemotactic migration of the sensor cells. This effective interaction through an attractant field provides an asymmetric coupling between the two cell populations, reminiscent of chemically active matter mixtures with nonreciprocal interactions (34).

After rescaling time and space by $t\to \eta t^{\prime }$, $x\to \sqrt{\eta D_{a}}x^{\prime }$ in Eqs. **1**–**3**, where $\eta ={\left(m{\overset{¯}{\rho }}_{c}\right)}^{-1}$ with a reference consumer density ${\overset{¯}{\rho }}_{c}$, and dropping the primes for clarity, we obtain the nondimensional system of equations

[4] $$\begin{array}{c} \partial_{t}\rho_{i}={\tilde{D}}_{i}\nabla^{2}\rho_{i}-{\tilde{\chi }}_{i}\nabla \cdot \left(\rho_{i}\frac{\nabla a}{a}\right)\;, \end{array}$$

and

[5] $$\begin{array}{c} \partial_{t}a=\nabla^{2}a-\rho_{c}a\;, \end{array}$$

where ${\tilde{D}}_{i}\equiv D_{i}/D_{a}$ and ${\tilde{\chi }}_{i}\equiv \chi_{i}/D_{a}$ are the rescaled diffusion and chemotactic coefficients. To fully specify the model, we solve the nondimensional system Eqs. **4** and **5** numerically on a 1D domain x∈[0,L] using finite difference methods with forward time stepping. We impose von Neumann boundary conditions with zero flux for both cell densities $\rho_{i}$ and the chemoattractant *a*. Initial conditions consist of a uniform attractant concentration and sharply localized cell density profiles at x=0 to focus on their patterning dynamics driven by self-generated gradients (see *SI Appendix*, section S1 for further details).

### Comigration of Cell Populations Is Regulated by Their Relative Chemotactic Response.

An immediate question arises when we consider the coupled migration dynamics of the chemotactic cell populations: Can the self-generated guidance field provide a robust mechanism for coordinated, long-range comigration beyond diffusive spreading? For a single population of consumer cells, a sufficiently large chemotactic coefficient ${\tilde{\chi }}_{c}$ can drive directed migration, as observed in various systems (2, 3, 5, 8). In line with these observations, we found that in the chemotactic regime with ${\tilde{\chi }}_{c}>{\tilde{D}}_{c}$, consumer cells propagated with a pronounced, though gradually broadening density peak (*SI Appendix*, Fig. S1*A*). This propagation is superdiffusive, with the mean position scaling as $\left\langle x_{c}\right\rangle \propto t^{\alpha_{c}}$ with an exponent $\alpha_{c}>0.5$ (*SI Appendix*, Fig. S1*B*), although the profiles do not form true traveling waves.

To explore the impact of the second, sensor cell population, we varied two key parameters: their i) rescaled diffusion constant ${\tilde{D}}_{s}$ and ii) rescaled chemotactic coefficient ${\tilde{\chi }}_{s}$. Changes in ${\tilde{D}}_{s}$ had little effect on the exact shape and position of the front (*SI Appendix*, Fig. S1*C*), so we fixed ${\tilde{D}}_{s}/{\tilde{D}}_{c}≃3$, reflecting experimental values for immune cells (see below), and focused on the role of ${\tilde{\chi }}_{s}$. Varying the relative strength of chemotaxis revealed the requirements for robust comigration: When ${\tilde{\chi }}_{s}<{\tilde{\chi }}_{c}$, sensor cells cannot maintain pace with the advancing consumer cell population and gradually lag behind, showing smaller mean positions $\left\langle x_{s}\right\rangle <\left\langle x_{c}\right\rangle$ and diminishing peak densities; see Fig. 1*B*. In the chemotactic regime of consumer cells (i.e. for ${\tilde{\chi }}_{c}>{\tilde{D}}_{c}$), we found a tightly coupled propagation pattern only when ${\tilde{\chi }}_{s}>{\tilde{\chi }}_{c}$, i.e. when the sensor cells have a stronger chemotactic sensitivity than the consumer cells. In this case, the sensor cells initially move faster but do not overtake the consumers indefinitely, as they reach regions with flat attractant gradients. This dynamic interplay leads to sustained sensor peaks localized ahead of the consumers; see Fig. 1*C*. For ${\tilde{\chi }}_{s}≳{\tilde{\chi }}_{c}$, the coupled comigration of the two cell populations was also reflected in their scaling exponents $\alpha_{c}≃\alpha_{s}$. This is in contrast with the uncoupled regime (${\tilde{\chi }}_{s}<{\tilde{\chi }}_{c}$), where the scaling exponents generically fulfill $\alpha_{s}<\alpha_{c}$. Importantly, we also checked that these features were conserved for the case ${\tilde{D}}_{s}<{\tilde{D}}_{c}$ (*SI Appendix*, Fig. S1*D*).

### Consumer-Sensor Separation Is Controlled by Chemotactic Sensitivity.

**Fig. 2.**
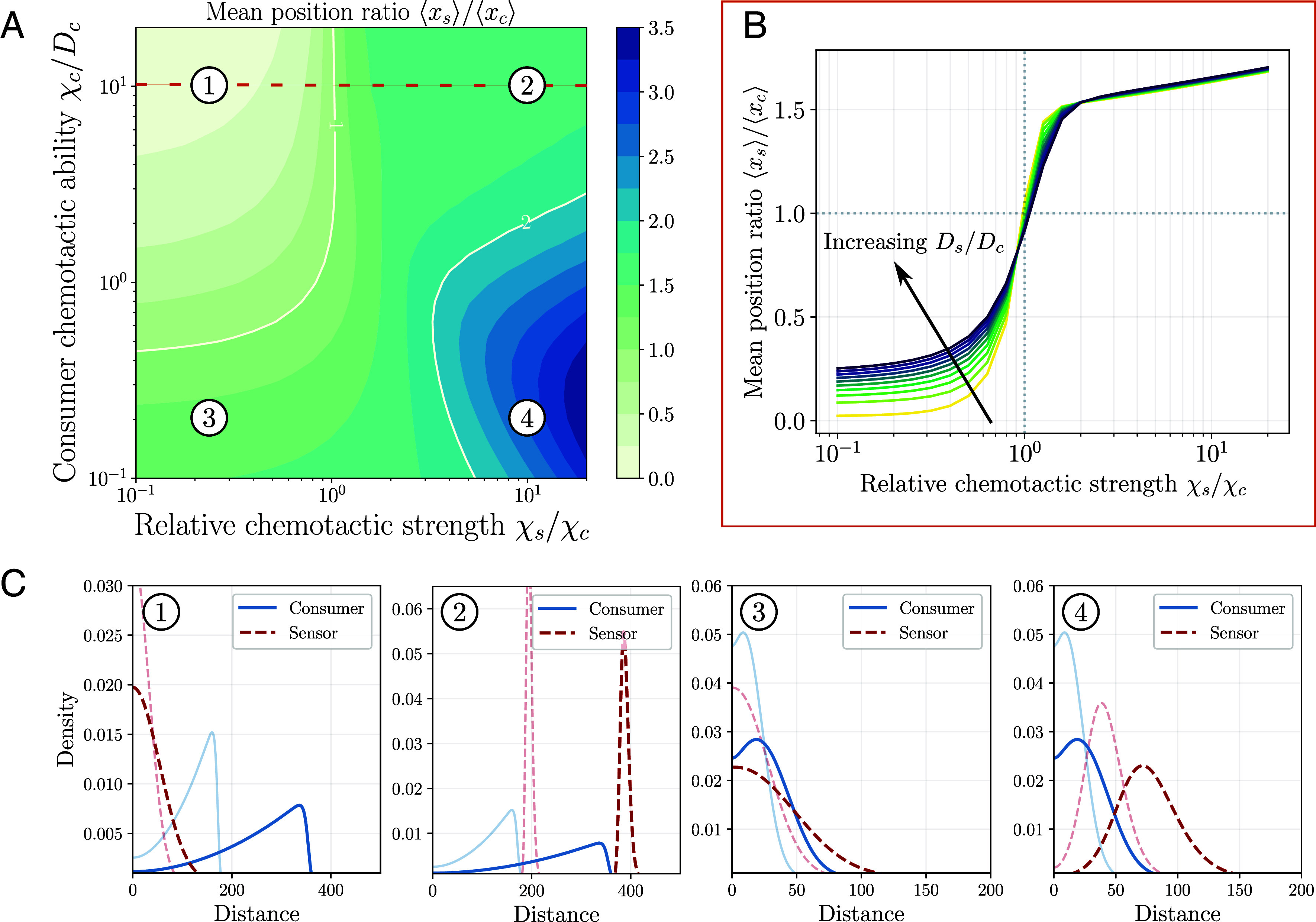
Relative positions and migration patterns of cell populations are controlled by their chemotactic sensitivity. (*A*) Long-time mean position ratio $\overset{¯}{x}\equiv \left\langle x_{s}\right\rangle /\left\langle x_{c}\right\rangle$ (color-coded) as a function of the rescaled chemotactic coefficient ratios ${\tilde{\chi }}_{s}/{\tilde{\chi }}_{c}$ and relative chemotactic prowess of consumer cells, as controlled by the ratio ${\tilde{\chi }}_{c}/{\tilde{D}}_{c}$. Dashed line (orange) corresponds to ${\tilde{\chi }}_{c}/{\tilde{D}}_{c}=10$ as plotted in (*B*). White contour lines outline the parameter regimes with $\overset{¯}{x}=1$ and $\overset{¯}{x}=2$. For consumer cells with a small ${\tilde{\chi }}_{c}/{\tilde{D}}_{c}$ ratio, the sensor cell position can exceed the bound $\sqrt{{\tilde{D}}_{s}/{\tilde{D}}_{c}}≃1.7$ dictated by diffusion as ${\tilde{\chi }}_{s}/{\tilde{\chi }}_{c}$ increases. (*B*) For sufficiently chemotactic consumer cells, e.g. with a chemotaxis-to-diffusion ratio ${\tilde{\chi }}_{c}/{\tilde{D}}_{c}=10$, the mean position ratio $\overset{¯}{x}$ of consumer and sensor cell population densities exhibits a bounded increase as a function of chemotactic coefficient ratio ${\tilde{\chi }}_{s}/{\tilde{\chi }}_{c}$, indicating that the sensor-cell position is controlled by consumer cells. Increasing the rescaled diffusion coefficient ${\tilde{D}}_{s}$ of the sensor cells (different colored lines) changes the ratio predominantly in the uncoupled regime for ${\tilde{\chi }}_{s}/{\tilde{\chi }}_{c}<1$. (*C*) Exemplary cell density profiles from different parameter regions plotted in (*A*) show the emergence of front- and pulse-like propagation patterns. Solid and dashed lines depict the profiles of consumer and sensor cells, respectively, at different time points (shaded lines).

Next, we wanted to understand more quantitatively how the separation between the two cell populations is regulated as a function of control parameters. Can for instance sensor cells that exhibit fast motility spread arbitrarily ahead of the consumer population? To explore this, we first systematically varied the chemotactic ability of the consumer cells ${\tilde{\chi }}_{c}/{\tilde{D}}_{c}$ and the relative chemotactic sensitivity ${\tilde{\chi }}_{s}/{\tilde{\chi }}_{c}$ of the two populations. Examining the long-time ratio of the mean position of cell densities $\overset{¯}{x}\equiv \left\langle x_{s}\right\rangle /\left\langle x_{c}\right\rangle$ displays several distinct regimes for the comigration dynamics: i) For sufficiently chemotactic consumer cells with ${\tilde{\chi }}_{c}/{\tilde{D}}_{c}>1$, weak sensors (${\tilde{\chi }}_{s}<{\tilde{\chi }}_{c}$) lead to uncoupled migration as discussed above, where the mean position ratio fulfills $\overset{¯}{x}<1$ (see Region 1 in Fig. 2*A*). Increasing the relative chemotactic strength ${\tilde{\chi }}_{s}/{\tilde{\chi }}_{c}$ leads to larger ratios, with a crossover of $\overset{¯}{x}=1$ at ${\tilde{\chi }}_{s}/{\tilde{\chi }}_{c}≃1$. However, in the coupled regime, the position ratio saturates for arbitrarily large ${\tilde{\chi }}_{s}/{\tilde{\chi }}_{c}$, highlighting that the sensor cell propagation is controlled by the consumers (see Region 2 in Fig. 2*A*). Interestingly, increasing the diffusion coefficient ratio ${\tilde{D}}_{s}/{\tilde{D}}_{c}$, i.e. the random motility of the sensor cells, is only beneficial for the sensor cell population in the uncoupled regime with ${\tilde{\chi }}_{s}/{\tilde{\chi }}_{c}<1$, while for ${\tilde{\chi }}_{s}/{\tilde{\chi }}_{c}>1$, it has a negligible effect; see Fig. 2*B*. ii) For weakly chemotactic consumer cells with ${\tilde{\chi }}_{c}<{\tilde{D}}_{c}$, the phase diagram displays two additional regimes: In the region ${\tilde{\chi }}_{s}/{\tilde{\chi }}_{c}<1$, the mean position ratio exhibits $\overset{¯}{x}>1$ while not exceeding the upper bound $\sqrt{{\tilde{D}}_{s}/{\tilde{D}}_{c}}≃1.7$ dictated by pure diffusion (see Region 3 in Fig. 2*A*). Indeed, for identical sensor and consumer diffusion coefficients, position ratio in this region remained within $\overset{¯}{x}<1$ (*SI Appendix*, Fig. S2*A*). For larger relative chemotactic sensitivities, i.e. for ${\tilde{\chi }}_{s}/{\tilde{\chi }}_{c}>1$, the position ratio can attain values exceeding $\sqrt{{\tilde{D}}_{s}/{\tilde{D}}_{c}}$, unlike the saturation behavior we found in the chemotactic regime ${\tilde{\chi }}_{c}/{\tilde{D}}_{c}>1$ (see Region 4 in Fig. 2*A*).

A closer look at the density profiles in the four regions allowed for more insight into the propagation dynamics: For sufficiently chemotactic consumer cells, the chemotactic response of the sensor cells is a key parameter to tune their density profile and relative position with respect to the consumer cell population, with a sharp transition from uncoupled to coupled comigration around ${\tilde{\chi }}_{s}≃{\tilde{\chi }}_{c}$. For large ${\tilde{\chi }}_{s}/{\tilde{\chi }}_{c}$ (e.g. Region 2) this coupling can then lead to the formation of a well-defined “sensor-cell pulse” ahead of the consumer cell front; see Fig. 2*C*. When the consumer cell population is in the weakly chemotactic regime (${\tilde{\chi }}_{c}/{\tilde{D}}_{c}<1$), the sensor cells can exhibit transient, pulse-like migration at the diffusive front of the consumers for large ${\tilde{\chi }}_{s}/{\tilde{\chi }}_{c}$ (Region 4). However, this peak rapidly decays as the chemoattractant gradient flattens along with the dispersing consumer population over time.

Time evolution of the density patterns further reveals that, across the entire phase space, including in the strong chemotaxis regime (Regions 1 and 2), the profiles decay slowly over time (see shaded lines in Fig. 2*C*), although their shapes are approximately scale-invariant (*SI Appendix*, Fig. S2*B*). In fact, the minimal system described by Eqs. **4** and **5** cannot exhibit traveling wave profiles in a strict sense (35) and the solutions will generically be “wave-like” profiles with slowly decaying amplitudes and velocities (36). The coupling behavior shown in Fig. 2 nevertheless persists over sufficiently large times because of the slow decay dynamics (see *SI Appendix*, Fig. S2*C* for a comparison across time points.). The absence of true traveling waves can be intuitively understood as follows: In the rear of the migrating front (i.e. for small *x*), the uptake term $-\rho_{c}a$ is dominated by a small attractant concentration *a*, which leads to a shallow gradient. For a conserved cell population, the chemotactic drift χ∇log(*a*) is then insufficient to counterbalance the diffusive leakage of cells in the back (37). Therefore, boundary conditions such as cell conservation in the system and attractant kinetics should directly influence the migration dynamics, as they modulate the cellular responses behind the front.

### Influence of Cell Influx on the Migration Dynamics.

**Fig. 3.**
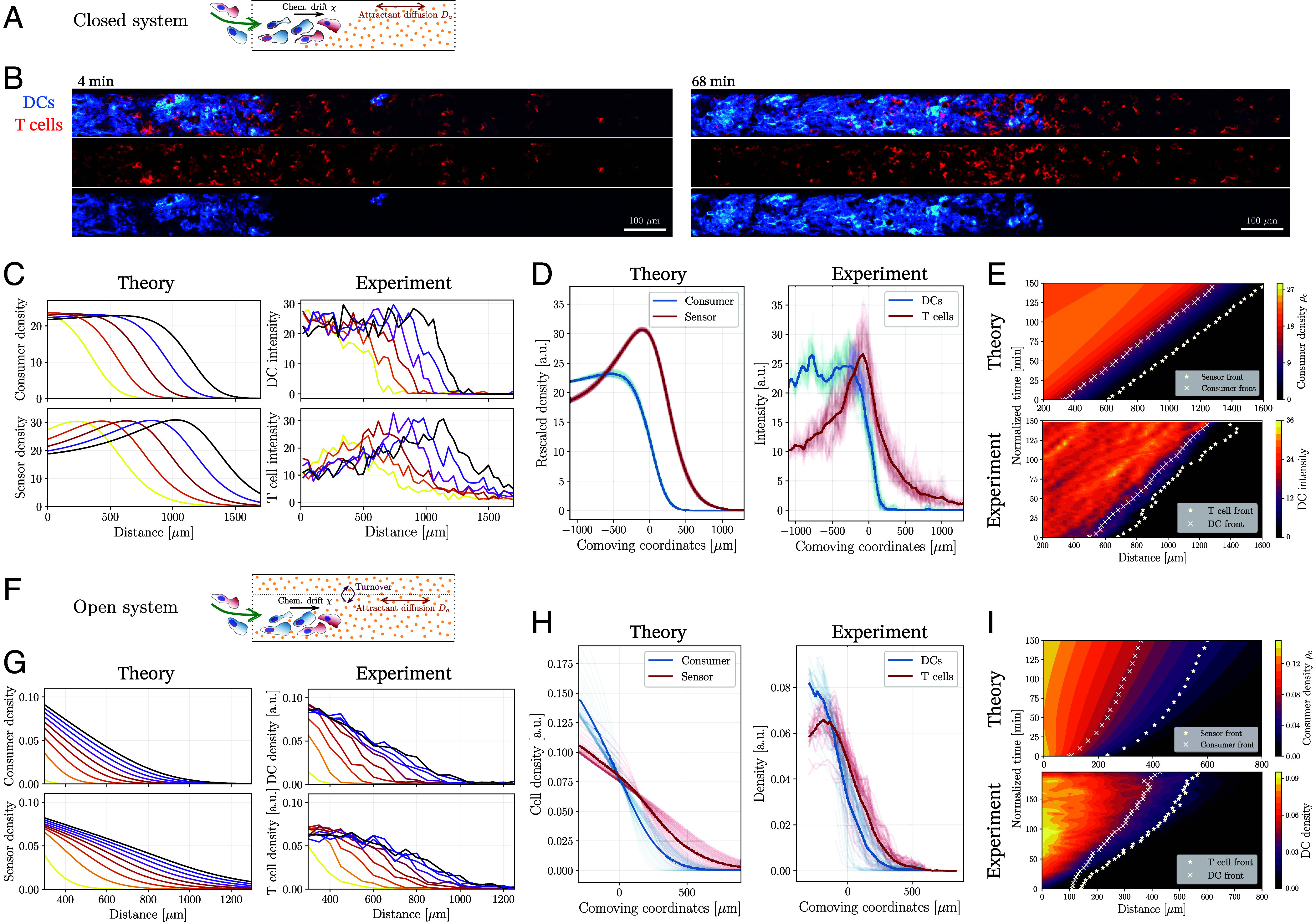
Comparison with in vitro experiments on collective migration of dendritic cells (DCs) and T cells. (*A*) Illustration of a *closed system*, where chemoattractant kinetics are described by its diffusion (with coefficient $D_{a}$) and interaction with the migrating cells that locally consume the attractant and experience a chemotactic drift. Cells enter the channel from the left boundary. (*B*) Microscopy images from the microfluidic channel experiments with labeled DCs (blue) and comigrating T cells (red) at t=4 min (*Left*) and t=68 min (*Right*). (Scale bar, 100μm.) (*C*) Spatiotemporal cell density profiles predicted by the analytical model (*Left*) agree well with the coupled propagation of front- and pulse-like densities as inferred from DC- and T cell intensities in experiments, respectively (*Right*). Color code indicates different time points. (*D*) Rescaled densities at different time points (shaded lines) overlaid in the reference frame comoving with the consumer- (*Left*) or DC (*Right*) front. Density profiles are qualitatively conserved over time with a well-defined separation between the fronts. (*E*) Kymographs showing the theoretical predictions on consumer migration (*Top*) and experimental data on DC migration (*Bottom*) over time. Cell density fronts are represented for both the consumer/DC (crosses) and the sensor/T cell (stars) population. Front positions over time indicate a constant speed of V≃5−6μm/min with which both cell populations migrate. (*F*) Illustration of an *open system*, where a chemoattractant reservoir (delineated by the dotted line) enables in- and efflux of attractant molecules, leading to an effective turnover kinetics. (*G*) Analytical model (*Left*) predicts spatiotemporal profiles without clear density peaks for both populations (*Left*), as observed in experiments (*Right*). (*H*) Cell densities in the comoving frame of the consumer (DC) front position display a nonconserved profile over time (shaded lines). (*I*) Kymographs showing the theoretical predictions on consumer migration (*Top*) and experimental data on DC migration (*Bottom*) over time. Density fronts are represented for both the consumer/DC (crosses) and the sensor/T cell (stars) population. Front positions over time indicate a slowly decaying speed both for theory and experiment. Experimental data in (*C*–*E*) represent averages from 5 independent experiments, and data in (*G*–*I*) from 4 experiments.

To explore the effect of external interactions on the migration dynamics in greater detail, we turned to a simple modification of our finite-difference numerical scheme by introducing a cell inflow into the system at the left boundary x=0, as illustrated in Fig. 3*A* (see *SI Appendix*, section S1 for numerical details). Strikingly, a small nonzero influx led to the formation of traveling waves with a constant velocity: The consumer cell density showed an initial density peak, which decayed into a wave front profile with a constant density at the back; see Fig. 3*C*, *Left* panel. In the coupled regime with ${\tilde{\chi }}_{s}>{\tilde{\chi }}_{c}$, the sensor cell profiles attained a density peak ahead of the consumer cells, with a conserved separation between the two cell fronts; see Fig. 3*D*, *Left* panel. Kymographs of cell densities showed that the velocities of the cell fronts (as quantified by the half-maximum of peak densities) were strictly matched with $V_{s}=V_{c}$ for ${\tilde{\chi }}_{s}\geq {\tilde{\chi }}_{c}$; see Fig. 3*E*, *Top* panel. When the sensor cell chemotactic coefficient was smaller than that of the consumer cells, however, sensor cells again “fell behind” the consumer cell population with a decaying speed and density profile (*SI Appendix*, Fig. S3 *A* and *B*). We also tested alternative scenarios in which only one cell type was introduced via boundary influx. Interestingly, we found traveling waves for both cell populations even when the boundary influx was applied solely to the consumer cell population. This suggests that consumer influx is sufficient for the efficient cotransport of sensor cells with a constant speed; see *SI Appendix*, Fig. S3 *E*–*H*.

### Comparison with Experimental Data on the Self-Organized Comigration of Immune Cell Populations.

To test our theoretical prediction on coupled migration patterns, we turned to a minimal experimental setup with heterogeneous immune cell populations. We focused on the collective migration of mature DCs and activated T cells that are terminally differentiated (see *Materials and Methods* for details), having recently observed qualitatively their comigration patterns mediated by the chemoattractant CCL19 (8). Biologically, DCs and T cells engage in close and repeated interactions in lymph nodes to trigger adaptive immune response. DCs both sense and deplete the chemoattractant CCL19, while T cells can sense but not significantly alter it, aligning with their roles as consumer and sensor cells, respectively. To closely match the coarse-grained description of the cell density and chemoattractant evolutions governed by Eqs. **4** and **5**, we designed a microfluidic channel system with two holes to control both the cell and attractant influx. Because there is no external chemoattractant reservoir in this setup, it represents a “closed” system in terms of attractant kinetics. After an equilibration period to allow for a uniform distribution of CCL19 in the channel, we introduced mixtures of DCs and T cells through one end. Both cell populations continued to enter the channel for over 2 to 3 h; see Fig. 3*B* and Movie S1.

Strikingly, we observed that the DC population formed a stable migrating front, while T cells accumulated ahead of this front, forming a distinct density peak as predicted theoretically; see Fig. 3*C*. To quantify this behavior, we located front positions based on the half-maximum of the smoothed cell density profiles (*SI Appendix*, Fig. S6*A*). When overlaying the profiles at different time points in a comoving frame centered on the DC front, both density profiles maintained their shape over time, with the two fronts consistently separated by a characteristic length scale of approx. 150 to 200 μm; see Fig. 3*D*. Furthermore, the kymographs obtained from the experiments confirmed the theoretical prediction on velocity matching between the two populations; see Fig. 3*E* and *SI Appendix*, Fig. S6*B*.

To test our prediction on the breakdown of velocity matching in the uncoupled regime (i.e. for the case ${\tilde{\chi }}_{s}<{\tilde{\chi }}_{c}$), we then looked at the comigration of DCs with CCR7-knockout (KO) T cells (which do not have the capacity to sense the ligand CCL19) in microfluidic channel experiments. We found that CCR7-KO T cells i) did not show any density peaks, ii) were not able to migrate ahead of the DC population, and iii) their speed was notably smaller than that of the DC front (*SI Appendix*, Fig. S7 *B*–*E* and Movie S2), as predicted theoretically for the uncoupled regime (*SI Appendix*, Figs. S3 *A* and *B* and S7*A*). Interestingly, even wild-type T cells exhibited a notable reduction in propagation speed when migrating alone, without DCs, either in a pre-established gradient or in a uniform CCL19 field (*SI Appendix*, Fig. S7*F* and Movie S3). This observation is fully consistent with our model, which predicts that sensor-only populations cannot sustain wave-like migration as they lack the ability to actively modulate the local steepness of the gradient.

### Prediction of the Front Velocity and Parameter Inference.

We then sought to constrain model parameters using two independent experimental setups. First, we analyzed DC migration trajectories from under-agarose assays without a chemoattractant (8). From this, we estimated the diffusion coefficient of DCs via both velocity autocorrelation analysis and Bayesian inference (*SI Appendix*, section S3), obtaining converging values around $D_{c}≃360\;\mu {\text{m}}^{2}/\;\text{min}$. Using our measured chemoattractant diffusion coefficient for CCL19 ($D_{a}≃86\;\mu {\text{m}}^{2}/\;\text{s}$) (8), this yielded a rescaled diffusion coefficient of ${\tilde{D}}_{c}=D_{c}/D_{a}≃0.07$ (*SI Appendix*, Fig. S5 *A*–*D*). Second, we used trajectories from the under-agarose assay for mixed populations of DCs and T cells with CCL19 and calculated the variance of velocity distributions for both populations, as the velocity fluctuations are proportional to their random motility coefficient. The comparison of this variance allowed us to approximate the ratio of their diffusion coefficients to be around $D_{s}/D_{c}≃3.5$ (see *SI Appendix*, Fig. S5*E* and section S3 for details).

Interestingly, the observation of a traveling cell front allowed us to theoretically predict the spatial shapes of the density profiles, as well as the propagation velocity V using simple arguments in the comoving frame of coordinates z≡x−Vt. Indeed, we predict that both the consumer and sensor cell populations should display exponentially decaying density profiles at the propagating edge with $\rho_{i}\propto \;\text{exp}\left(-\zeta_{i}z\right)$, with a decay length $\zeta^{-1}$ related to the front velocity by $V\propto D_{i}\zeta_{i}$. This relation means that the ratio of decay lengths is simply proportional to the ratio of diffusion coefficients between the consumer and sensor cell populations, i.e. $D_{s}/D_{c}=\zeta_{c}/\zeta_{s}$, which we confirmed numerically (*SI Appendix*, Fig. S3*C*). Importantly, we then used this relation to compare the experimental density profiles of DC (consumer) and T cell (sensor) populations in the comoving frame, and from their decay lengths obtained a value of $D_{s}/D_{c}≃2.7$ (*SI Appendix*, Fig. S5*F*), closely aligning with independent estimates described above.

Furthermore, we numerically observed that the consumer cells exhibit a spatially flat profile behind the front with a constant density $\rho_{c}^{†}$, with the chemoattractant concentration showing an exponential decay approximated by a∝exp(λz) in the rear of the propagating front. Using this information, we integrated the system of equations in the comoving frame and found that the decay length scale of the chemoattractant and the front velocity are linked by the relation $V=\chi_{c}\lambda$. In dimensional units, we could then obtain a simple expression for the front velocity (see *SI Appendix*, section S1 for the derivation):

[6] $$\begin{array}{c} V=\chi_{c}\;\sqrt{\frac{m\rho_{c}^{†}}{D_{a}+\chi_{c}}}\;. \end{array}$$

This relation suggests that the front velocity scales as $V\propto \chi_{c}^{0.5}$, and depends on an effective time scale of chemoattractant internalization $\tau ∼{\left(m\rho_{c}^{†}\right)}^{-1}$ fixed by the rate *m* and bulk consumer density $\rho_{c}^{†}$, while similar expressions have been found under different assumptions (25, 28). We then tested this prediction for the front velocity numerically by varying the chemotactic coefficient $\chi_{c}$ and found good agreement with Eq. **6** (*SI Appendix*, Fig. S3*D*).

We next aimed to infer the chemotactic response functions directly from experimental cell density profiles. By leveraging the total flux balance in the comoving frame, we decomposed mass transport into diffusive and chemotactic components. This allowed us to reconstruct the response function directly from the cell density profiles and their gradients, without requiring knowledge of the attractant field. The inferred chemotactic responses were consistent with a relative sensing form, exhibiting a nearly constant chemotactic drive in the back of the wave (*SI Appendix*, Fig. S6 *D* and *E*). By taking the ratio of inferred responses for sensor and consumer populations, we further obtained a quantitative estimate for the relative chemotactic strength $\chi_{s}/\chi_{c}≃1.26\pm 0.2$, a regime predicted by the model to enable robust comigration (see *SI Appendix*, Fig. S6*F* and section S3 for full methodological details).

We then used a sufficiently large chemotaxis-to-diffusion ratio of ${\tilde{\chi }}_{c}/{\tilde{D}}_{c}≃3$ for consumer cells to obtain a well-defined wave front, and fitted the timescale $\eta ={\left(m{\overset{¯}{\rho }}_{c}\right)}^{-1}≃0.1\;\;\text{min}$ which provided a good quantitative agreement between theory and experiment for several distinct features (as displayed in Fig. 3): i) the scaling of the density profiles at both front and back agreed closely with the experimental data, ii) the predicted separation between the two populations was ∼250 μm, compared to ∼170 μm in experiments, and iii) the predicted front velocity V≃5−7μm/min quantitatively matched the experimental front speeds. Finally, we used the aforementioned values for $\chi_{c}$, $D_{a}$, and only by fitting the time scale ${\left(m\rho_{c}^{†}\right)}^{-1}≃5\;\;\text{min}$ in Eq. **6**, we obtained an analytical estimate of V≃5.8μm/min, in close agreement with both experiment and the numerical results. Parameter scans furthermore confirmed that the density patterns were robust to variations in diffusion and chemotactic coefficients within their error ranges, identifying the relative chemotactic strength $\chi_{s}/\chi_{c}$ as the key parameter controlling comigration behavior (*SI Appendix*, Fig. S7*A*).

### Influence of Chemoattractant Turnover on the Migration Dynamics.

Next, we asked whether modifying the chemoattractant kinetics might influence the migration patterns in addition to the boundary influx of cells. Indeed, Eq. **5** suggests that a turnover of attractant density, e.g. arising from an external reservoir, should directly influence the dynamics of the gradient. We thus implemented a minimal model for the attractant kinetics to describe additional decay and influx terms; see Fig. 3*F* for an illustration and *SI Appendix*, section S1 for details. We found that numerically solving this “open system” led to strikingly different migration patterns: First, density profiles exhibited monotonically decaying shapes without well-defined peaks in a large region of the parameter space. In fact, sensor cell “pulses” only existed for relatively large chemotactic coefficients and consumer cells never exhibited pulse-like density peaks (*SI Appendix*, Fig. S8). Second, we could not find any parameter regime that led to the formation of traveling waves. Density profiles generically evolved with slowly decaying velocities. Third, even for very large chemotactic coefficients, the long-time front propagation dynamics showed diffusive scaling exponents around α≃0.5, unlike the traveling waves of the closed system. The absence of traveling waves with attractant turnover presumably arises from the restoring effect of turnover, which drives the attractant profile toward uniformity and flattens self-generated gradients.

To test these theoretical signatures, we turned to experimental data on the collective migration of DCs and T cells in the under-agarose assay, as we had qualitatively explored before (8). In this system, a large reservoir of the chemoattractant CCL19 is in constant contact with the confined quasi-2D space where the cells migrate. Plotting the cell density profiles over time, we found that the densities exhibited monotonically decaying profiles as predicted by the theory; see Fig. 3*G* for a comparison. Overlaying the profiles at different times, we also found that T cells migrated ahead of the DC population at all times with a length scale of the same order of magnitude as in the microfluidic channel, see Fig. 3*H*, but with an increasing separation of cell fronts over time, see Fig. 3*I*. This behavior matches with the theoretical prediction using a chemotactic strength ratio of ${\tilde{\chi }}_{s}/{\tilde{\chi }}_{c}≃1.2$ that we had previously estimated for the closed system, although their absolute values in the open system are larger. Finally, the long-time propagation dynamics of density fronts showed that both populations migrated with decaying velocities over time, as represented in the kymographs in Fig. 3*I*. Overall, the analysis of this minimal open system indicates that attractant kinetics can indeed lead to radically different migration dynamics for chemotactic cell populations.

### Relative Chemotactic Sensitivity Controls Colocalized Migration of Cell Populations.

**Fig. 4.**
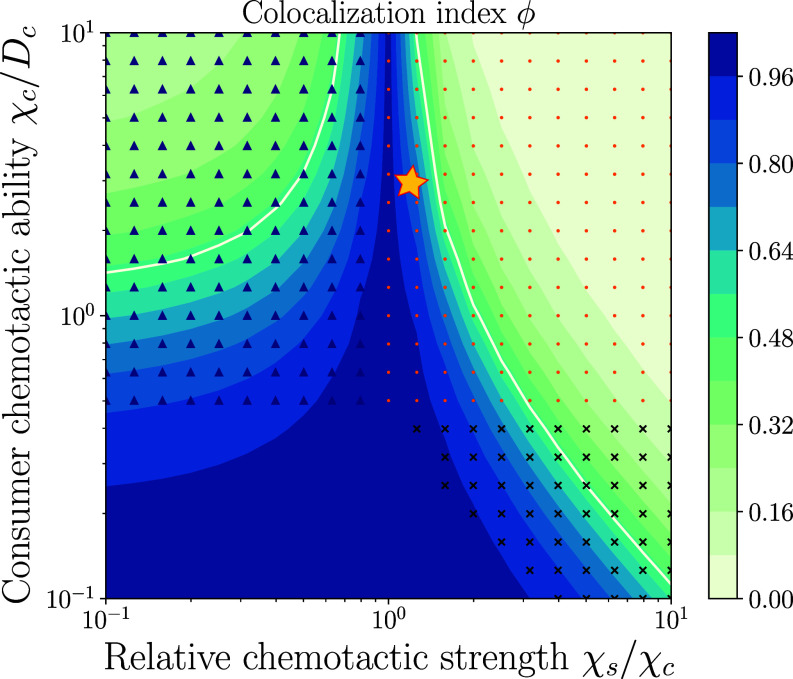
Phase diagram of colocalization of different cell populations indicates an optimal regime for coupled migration. Colocalization index (color-coded) defined as $\phi \equiv 1-D_{\mathrm{JS}}$, using the Jensen–Shannon divergence ($D_{\mathrm{JS}}$) of density distributions for sensor and consumer cells. Regions with ϕ>0.5 indicate sensor cells being transported by consumers with enhanced colocalization. White contour lines depict ϕ=0.5. For sufficiently chemotactic consumer cells, i.e. for ${\tilde{\chi }}_{c}/{\tilde{D}}_{c}>1$, the colocalization index displays a nonmonotonic behavior with increasing ${\tilde{\chi }}_{s}/{\tilde{\chi }}_{c}$, with an optimal region for $1\leq {\tilde{\chi }}_{s}/{\tilde{\chi }}_{c}\leq 2$. Parameter values used for the comparison with the experiments are ${\tilde{\chi }}_{c}/{\tilde{D}}_{c}≃3$ and ${\tilde{\chi }}_{s}/{\tilde{\chi }}_{c}≃1.2$ (star symbol). Distinct regions of density patterns are highlighted with markers for i) only pulse-like consumer cells (blue triangles), ii) pulse-like consumer and sensor cells (orange dots), iii) no peaks (no markers), and iv) only pulse-like sensor cells (black crosses). Rescaled diffusion coefficients used here are ${\tilde{D}}_{c}={\tilde{D}}_{s}=0.1$.

Having explored the coupled migration regimes in different setups, we then wished to go back to the minimal system described by Eqs. **4** and **5** and ask whether we could quantitatively measure the degree of overlap or colocalization between distinct populations. In addition to comigration, the question of colocalization of heterogeneous cell populations is important particularly to explore conditions for possible physical interactions between different immune cell types, as they need to communicate in close proximity during an immune response (38). To quantify this, we first introduced a colocalization index using the Jensen–Shannon divergence (JS) of spatial density profiles interpreted as probability densities (see *SI Appendix*, section S5 for details). We defined a simple metric for similarity or overlap of cell densities as $\phi \equiv 1-D_{\mathrm{JS}}$, where $D_{\mathrm{JS}}$ denotes the Jensen–Shannon divergence. With this definition, ϕ=1 corresponds to complete colocalization while ϕ=0 indicates zero overlap of densities. Interestingly, this simple metric already outlined two distinct regions in the phase space where efficient consumer migration occurs (for sufficiently large ${\tilde{\chi }}_{c}/{\tilde{D}}_{c}≳1$), but with impaired colocalization; see Fig. 4. Intuitively, in the uncoupled regime (${\tilde{\chi }}_{s}/{\tilde{\chi }}_{c}<1$), colocalization is small as the sensor cells in this case fall behind the propagating consumer front. However, in the strongly coupled regime (${\tilde{\chi }}_{s}/{\tilde{\chi }}_{c}>2$), the sensor density peak is too far ahead of the consumers, which also results in minimal density overlap. Furthermore, while random cellular diffusion (controlled by ${\tilde{D}}_{i}$) is detrimental for robust chemotactic front propagation, it is in fact beneficial for colocalization, as very small diffusion coefficients result in very sharp density peaks, and thus small density overlaps. This analysis thus predicted that each parameter of the system exhibited trade-offs, while acting to optimize for both comigration and colocalization. Notably, the values which fitted best our experimental data were located in the “optimal” region in the coupled regime of the phase diagram, where $1\leq {\tilde{\chi }}_{s}/{\tilde{\chi }}_{c}\leq 2$ and ${\tilde{\chi }}_{c}/{\tilde{D}}_{c}>1$; see Fig. 4 (star symbol). Finally, while we used identical diffusion coefficients for sensors and consumers in Fig. 4, changing to experimentally inferred parameter estimates for the diffusion coefficients only modified the colocalization in the diffusively uncoupled regime, leaving the remaining phase diagram unchanged (*SI Appendix*, Fig. S9).

### Influence of Nonreciprocal Mechanical Interactions.

We next examined how mechanical interactions between cell populations, especially nonreciprocal ones where, for instance, population A repels B but not vice versa, affect chemotactic comigration (39, 40). We extended our Keller–Segel model by including linear advective coupling terms between the two populations:

[7] $$\begin{array}{c} \partial_{t}\rho_{i}=D_{i}\nabla^{2}\rho_{i}-\chi_{i}\nabla \cdot \left(\rho_{i}\nabla a/a\right)+\mu_{i}\nabla \cdot \left(\rho_{i}\nabla \rho_{j}\right)\;, \end{array}$$

with i≠j, and $\mu_{i}$ represents the mechanical advection parameter for the *i*-th cell population (i=c for consumers, i=s for sensors). Positive values (μ>0) indicate repulsion (cells being “pushed”), while negative values (μ<0) represent attraction (cells being “pulled”); see *SI Appendix*, section S6 for details.

Numerical solutions showed that the front offset between populations, quantified by the mean position ratio $\overset{¯}{x}=\left\langle x_{s}\right\rangle /\left\langle x_{c}\right\rangle$, was strongly modulated by mechanical interaction asymmetry $\Delta \mu =\mu_{s}-\mu_{c}$, especially when consumer chemotaxis was weak ($\chi_{c}/D_{c}<1$). Here, large $\mu_{s}$ and small $\mu_{c}$ enhanced $\overset{¯}{x}$, while stronger chemotaxis suppressed this effect (*SI Appendix*, Figs. S10 *A* and *B* and S11).

Furthermore, we found that mechanical interactions could either enhance or disrupt coordinated migration depending on whether they are attractive or repulsive. When diffusively migrating sensors strongly repelled consumers ($\mu_{c}>0$), they effectively “pushed” consumers forward, which increased their separation from sensor densities. This repulsion reduces the consumers’ ability to sense the attractant gradient efficiently. Conversely, for strong sensor chemotaxis, consumers can be “pulled from ahead” for $\mu_{c}<0$, resulting in a broader density profile that locally flattens the attractant gradient (*SI Appendix*, Fig. S10 *C*–*E*). These findings thus highlight the system’s inherent nonreciprocity and the role of mechanical interactions modulating the comigration patterns in regimes of weak or intermediate consumer chemotaxis.

## Discussion

Self-generated chemoattractant gradients have recently emerged as a robust, self-organized mechanism for long-range guidance of cellular collectives (10). Here, we propose a theoretical framework showing how this mechanism can also coordinate heterogeneous cell mixtures without direct cell–cell interactions. We tested several key predictions of the model experimentally using comigrating dendritic cells (DCs) and T cells. Our model quantitatively reproduces essential features of their self-generated guidance, including the spatial density profiles, relative speeds, and the influence of external chemoattractant reservoirs on the dynamics. It also reveals qualitatively distinct modes of comigration and highlights trade-offs in key parameters. For example, sensor cells must exhibit stronger chemotaxis than consumer cells to stay with the propagating front, typically resulting in a density peak of sensors ahead of consumers. However, excessive chemotactic strength of sensors leads to poor colocalization with consumers. Similarly, while random motility reduces chemotactic efficiency, it increases the chance of colocalized migration. Notably, direct (nonreciprocal) mechanical interactions between cell types become relevant only when chemotaxis is weak.

Biologically, DC and T cell interactions are central to adaptive immunity and occur within the lymph nodes, where both cell types are guided using the chemokine receptor CCR7 to sense chemoattractants like CCL19 produced by stromal cells (41). These encounters are not singular events but involve a dynamic crosstalk, where cells repeatedly meet and scan each other to integrate signals on the population level (42). Similar multicellular streams have also been observed, for example, in CCR7-dependent trafficking of T cells in zebrafish (43) and their directional crawling between two compartments in the mouse spleen. In both scenarios, it seems unlikely that a fixed spatial gradient could guide the cells over distances of many millimeters. Our model proposes a simple mechanism by which a self-generated chemoattractant field facilitates sustained interactions and coordinated migration of these mixed cell populations, consistent with observations of self-generated signaling gradients in the developing lateral line during zebrafish development (2, 44).

Collective long-range guidance of cell populations can arise from various local interactions, including cell–cell repulsion, contact inhibition of locomotion, polarity alignment (45), and mechanochemical coupling (46). A range of modeling approaches, such as vertex and Voronoi models (47, 48), phase-field models (49), continuum models based on nonlocal sensing (50), and hybrid models incorporating cell-intrinsic states (51), have been developed to study the emergence of collective directionality. However, how heterogeneous cell populations with distinct attractant-shaping capabilities organize and coordinate their comigration remains an open question (30). In this context, our multi-component Keller–Segel model could be extended to include interconversion between sensor and attractant-modulator types, in line with recent work on phenotypic plasticity (30, 52, 53).

While some previous models rely on a leader–follower mechanism for directional migration (17, 54), recent experimental evidence suggests that many cell types, including neural crest (55), immune (8) and cancer cells (5), migrate efficiently without fixed leaders. Moreover, in epithelial cell clusters, local mechanical pulling by leader cells appears insufficient to drive collective migration (56). Interestingly, in a leader-follower paradigm, T cells could be seen as followers as they receive guidance cues from DCs, yet they are migrating ahead of the DC front, emphasizing the effect of indirect, gradient-mediated guidance. Our model supports a simple paradigm in which distinct cell types remain fully motile and responsive without direct contact, achieving efficient comigration through adaptive gradient sensing alone.

Beyond chemoattractants, other types of self-generated gradients based on substrate rigidity or extracellular matrix remodeling have emerged in recent years (57–59). Like chemotactic systems, these involve cells modifying their environment to encode a memory of past trajectories, leading to emergent directionality. Although mechanical cues are typically more localized than diffusible signals, the core features of our model are expected to extend to such contexts. Crucially, interactions between sensors and consumers are intrinsically nonreciprocal due to their asymmetric coupling via the attractant, directly linking our work to recent advances in synthetic active matter, including chemically active mixtures (34, 60) and cross-diffusive systems with nonreciprocal interactions (61). Our results may thus inform both synthetic systems and experimental studies, such as in vitro motility assays (13) and live imaging of heterogeneous cell migration (62).

## Materials and Methods

### Cell Culture.

All cells used in this study were grown and maintained at +37C with 5% CO_2_ in a humidified incubator.

### Dendritic Cell Differentiation and Maturation.

DCs were generated from previously described LifeAct-GFP expressing conditionally HoxB8 immortalized hematopoietic progenitor cells (63, 64). LifeAct-GFP Hoxb8 cells were maintained in R10 medium [RPMI 1640 medium (Gibco, 21875-034) with 10% heat-inactivated fetal bovine serum (Gibco, 10270-106), penicillin (100 U/ml)/streptomycin (100 μg/ml) (Gibco, 15140-122), and 50 μM *β*-mercaptoethanol (Gibco, 31350-010)] supplemented with 5% supernatant of an Flt3L-producing cell line and 1 μM estrogen (Sigma-Aldrich, E2758). For DC differentiation, $3\times 10^{5}$ Hoxb8 precursor cells were cultured in 10 ml R10 supplemented with 20% of house-generated Granulocyte-macrophage colony stimulating factor (GM-CSF) hybridoma supernatant. On day 3 of differentiation, additional 10 ml R10 medium supplemented with 20% GM-SCF were added to the dish, on day 6 followed by replacement of half of the R10 medium with 20% GM-SCF. On day 9 DCs were harvested for maturation. DC maturation was induced by overnight incubation with lipopolysaccharide (LPS, 200 ng/ml) from *Escherichia coli* 0127:B8 (Sigma-Aldrich, L4516).

### T Cells.

T cells were isolated from spleens of male mTmG reporter (65) and CCR7 deficient mice (66) using the EasySep™ Mouse T Cell Isolation Kit (STEMCELL Technologies, 19851) following manufacturers’ instructions and activated with anti-mouse CD28 (Invitrogen, 16-0281-85) and CD3e (Invitrogen, 16-0031-85) 1 μg/ml coated to cell culture dishes. Activated T cells were cultured in R10 medium substituted with 10 ng/ml IL-2 (PeproTech, 212-12) for up to 14 d. Before migration assays CCR7 deficient T cells were stained with TAMRA (Invitrogen) 1:1,000 in PBS for 10 min at room temperature, washed 2× with PBS right before the assay.

### Migration Assays in Microfabricated Channels.

Microfabricated polydimethylsiloxane (PDMS) devices were prepared as described previously (8). Fabricated PDMS devices with straight channels (300 × 100 × 4,5 μm) were flushed with R10 medium. For uniform chemokine assay, one of the reservoirs in the PDMS device was refilled with 1,25 ng/μl of CCL19 (R&D Systems, 440-M3-025) supplemented R10 medium and allowed to equilibrate for 2 to 3 h at 37 ^°^C and 5% CO2. After the incubation, 2 μl of pelleted DC and T cell mixture was added to the opposite side reservoir in the PDMS device. For T cell migration in chemokine gradient, 2 μl of pelleted T cells were added at one side of the device and 1,25 ng/μl CCL19 in R10 medium to the opposite side at the same time. The time-lapse imaging of migrating cells was performed by imaging every 30 s with an inverted Nikon Ti2E wide-field fluorescent microscope at 37 ^°^C with 5% CO2 for the duration of 4 to 6 h, using Nikon 10× objective (Plan Fluor 10×/0.3 DIC 1 N1 air PFS).

## Supplementary Material

Appendix 01 (PDF)

Movie S1. Collective Migration of DCs and T cells.

Movie S2. Collective Migration of DCs and CCR7 KO T cells.

Movie S3. T cell migration in uniform CCL19.

## Data Availability

Custom-made scripts have been deposited in GitHub (https://github.com/mehmetcanucar/Self-generated-chemotaxis) (67).
