# Supplementary material for: Self-generated chemotaxis of mixed cell populations
Source: Proc Natl Acad Sci U S A. 2025 Aug 21;122(34):e2504064122. doi: 10.1073/pnas.2504064122 (PMC12403085; doi:10.1073/pnas.2504064122)
Supplement: Supplementary file 1 — Appendix 01 (PDF) [file pnas.2504064122.sapp.pdf]

Supplementary Information - Self-generated chemotaxis of mixed cell populations

Mehmet Can Uçar, Zane Alsberga, Jonna Alanko, Michael Sixt, Edouard Hannezo

June 12, 2025

Contents

S1 Details on the coarse-grained model **2**

S1.1 Details on numerical solution of the PDEs 3

S1.2 Analytical prediction for the traveling wave velocity 4

S1.3 Chemoattractant kinetics in closed vs. open systems 5

S2 Model predictions and sensitivity analysis **6**

S2.1 Effects of parameter variations and temporal evolution 6

S2.2 Influence of model assumptions on traveling wave properties 9

S3 Parameter estimates, model fitting and perturbation experiments **12**

S3.1 Inference of diffusion coefficients 13

S3.1.1 Diffusion coefficient of consumer/dendritic cells 13

S3.1.2 Diffusion coefficient of sensor / T cells. 16

S3.2 Inference of chemotactic sensing functions and relative sensing strength χ_s/χ_c 17

S3.3 Parameter fitting and model robustness 19

S3.4 Co-migration of dendritic cells and CCR7-KO T cells 21

S3.5 T cell migration in uniform and imposed gradients 21

S4 Migration patterns in the open system **22**

S5 Jensen-Shannon divergence for colocalization **23**

S6 Mechanical interactions between cell populations **25**

S7 List of Supplementary Movies **28**

In this Supplementary Note, we provide additional details on the modelling approach, analytical predictions, parameter inference, numerical solution methods, as well as sensitivity analyses on how different model setups and parameter choices affect our results.

S1 Details on the coarse-grained model

Continuum modeling approaches for chemotaxis can be generically formulated in the framework of a persistent and biased random walk, as it was introduced by Patlak [1]. Different microscopic mechanisms based on Fick's law, "space-jump" processes, or transport equations can be used to obtain coarse-grained continuum models for chemotaxis [2]. These different derivations lead to coupled PDE systems, as analyzed by Keller and Segel [3, 4], which can result in spatial patterning when the cells both produce and migrate up chemoattractant gradients. Although the original Keller-Segel system involved a single chemoattractant-modulating cell population together with the dynamics of the attractant, it is rather straightforward to introduce additional chemotactic cell populations in this framework. One can then ask whether the different cell types will be governed by a symmetric description in terms of (i) their chemotactic sensing/response, which controls their advective speed, and/or (ii) their interactions with the chemoattractant concentration, i.e. whether they act as producers or consumers of the attractant. In the context of bacterial chemotaxis, for instance, such *heterogeneity* in chemotaxis has been studied with respect to the chemotactic responses of different cell types [5, 6, 7, 8]. However, heterogeneity in terms of attractant modulation has remained largely unexplored. Here, we will focus on a Keller-Segel framework to describe mixed cell populations with distinct roles where we have *consumer-sensor* cells that both sense and shape, and *sensor* cells that can only sense attractant gradients. The simplest formulation of the coupling between these two cell populations via the chemoattractant is then defined by

$$\partial_t \rho_i = D_i \nabla^2 \rho_i - \nabla \cdot (\rho_i \mathbf{v}_i), \tag{S1}$$

with the drift velocity $\mathbf{v}_i \equiv \chi_i \nabla \log(a) = \chi_i \nabla a / a$, and the subscript $i = c, s$ describes the consumer or sensor population, respectively. The chemoattractant profile will be governed by diffusion with coefficient D_a and its consumption by the consumer population with rate m:

$$\partial_t a = D_a \nabla^2 a - m \rho_c a, \tag{S2}$$

Note that the chemotactic drift velocity $\mathbf{v} = \chi \nabla \log(a)$ represents the simplest formulation of a Weber-Fechner type of attractant sensing (relative sensing), as discussed originally by Keller and Segel. Alternative formulations for the sensing are possible for instance by considering (i) absolute sensing of the gradient, i.e. $\mathbf{v} = \chi \nabla a$, (ii) assuming an upper sensing threshold K with Michaelis-Menten kinetics, i.e. $\mathbf{v} = \chi \nabla a/(a+K)$ [9], or (iii) considering a bounded logarithmic sensing regime within lower and upper attractant concentrations K_- and K_+, respectively, i.e. $\mathbf{v} = \chi \nabla \log[(1+a/K_-)/(1+a/K_+)]$ [6, 10, 11]. For small K_- or small K, both the bounded logarithmic sensing and the Michaelis-Menten sensing will converge to $\nabla a/a$. In general, if the drift velocity vanishes quickly as $a \to 0$, e.g. for absolute sensing, Michaelis-Menten kinetics with large K, or bounded

logarithmic sensing with large K_-, we find that the migration patterns become less chemotactic and traveling waves cannot be formed, see the discussion in Section S2 below.

As explained in the main text, we can nondimensionalize the Eqs.(S1-S2) by $t \to (m\bar{\rho}_c)^{-1}t'$ and $x \to \sqrt{\frac{D_a}{m\bar{\rho}_c}}x'$, with a reference cell density $\bar{\rho}_c$. This spatiotemporal rescaling then leads to the nondimensional coupled PDE system:

$$\partial_t \rho_i = \tilde{D}_i \nabla^2 \rho_i - \tilde{\chi}_i \nabla \cdot \left(\rho_i \frac{\nabla a}{a} \right), \tag{S3}$$

and

$$\partial_t a = \nabla^2 a - \rho_c a, \tag{S4}$$

where $\tilde{D}_i \equiv D_i/D_a$ and $\tilde{\chi}_i \equiv \chi_i/D_a$ are the rescaled diffusion and chemotactic coefficients.

S1.1 Details on numerical solution of the PDEs

To solve the coupled nondimensional PDE system in 1D (as given by Eqs.(S3-S4) and (S12-S13)), we use the finite difference method to approximate the spatial and temporal derivatives. In particular, we discretize spatial coordinates within boundaries $0 \le x \le L$ in $N+1$ intervals of equal size Δx such that $x_n = n\Delta x$, with $n = 0, \dots, N$. Similarly, we discretize time in intervals of size Δt such that $t_k = k\Delta t$. For the time derivatives, we use the forward difference approximation such that $\partial_t \rho(x,t) \simeq (\rho(x_n, t_k + \Delta t) - \rho(x_n, t_k))/\Delta t$. We furthermore use von Neumann boundary conditions with $\partial_x \rho(x = 0, t) = \gamma$ and $\partial_x \rho(x = L, t) = \eta$, where γ and η define the fluxes through the right and left boundaries, respectively. As evaluating the second derivative at $x_0 = 0$ requires a value for $x^* = -\Delta x$ in the centered difference method, we introduce an additional lattice point for $n = -1$ and evaluate $\partial_x^2 \rho(0,t) \simeq 2(\rho(x_1, t_k) - \rho(x_0, t_k) - \gamma\Delta x)/\Delta x^2$ as an approximation at the boundary $x_0 = 0$. Likewise, the right boundary condition then dictates $\partial_x^2 \rho(N,t) \simeq 2(\rho(x_{N-1}, t_k) - \rho(x_N, t_k) + \eta\Delta x)/\Delta x^2$, where $\partial_x \rho(x = L, t) = \eta$. In all cases studied here, we will assume that there is no influx at the right boundary such that $\eta = 0$.

To analyze the core features of the model (as given in Figs.1, 2 and 4 in the main text) we use no-flux boundary conditions with $\gamma = \eta = 0$. To simulate cell influx into the system and reproduce the experimental setup, we modified the left boundary using two alternative methods: First, we injected a small increment of cells at $x_0 = 0$ by applying $\rho(x = 0, t + \Delta t) = \rho(x = 0, t) + \varepsilon\Delta t$, where ε is the rate of cell inflow. As an alternative method, we modified the boundary derivative directly by setting a small nonzero $\gamma > 0$ at $x_0 = 0$. Physically, this corresponds to a diffusive influx of cells into the system. We found that both methods gave almost identical traveling wave patterns for sufficiently small influx rates, and used the direct cell injection method for the comparison with experimental data (as shown in Fig.3 of the main text).

As we focus on chemotaxis driven by self-generated attractant gradients, we set the initial chemoattractant concentration to be uniformly distributed over space with a constant value, which we take for simplicity to be $a(x, t = 0) = 1$. We then initialize the consumer and cell density profiles to have sharply decaying profiles localized at $x = 0$, with initial values determined by $\rho_i(x, t = 0) = 1/(1 + A\exp(x - B))$, where A and B can be tuned to control the decay strength of the initial density profiles. We typically set $A = B = 1$, except for

simulations matching the open-system experiments, where we chose $A = 1$ and $B = 5$. We tested that different choices with sharply localized and strongly decaying profiles in general lead to similar migration dynamics, including e.g. using Gaussian density profiles with a small variance. Once initial conditions are defined, we recursively update the cell and attractant profiles as determined by the finite difference scheme until a sufficiently large time $T \propto 10^3 - 10^4$ is reached. For numerical stability, we set the time step to be $\Delta t = 0.01$ and the spatial resolution to $\Delta x = 1$.

S1.2 Analytical prediction for the traveling wave velocity

For the system with cell influx, we numerically find a traveling wave-like propagation of cell density profiles with a well-defined velocity. Here we derive a simple analytical expression to estimate the velocity of this traveling wave front. For coupled consumer and sensor cell populations, i.e. for $\chi_s \geq \chi_c$, this velocity is controlled solely by the consumer cells that locally shape the attractant gradient. We can therefore aim to find this velocity by considering the front-like propagation of the consumer cells and the attractant dynamics. To seek for self-similar density profiles in time, we switch to a comoving frame $z \equiv x - \mathscr{V}t$ with the front speed $\mathscr{V}$, and rewrite Eqs.(S1-S2) as:

$$-\mathscr{V}\rho_c' = D_c\rho_c'' - \chi_c\left(\rho_c\frac{a'}{a}\right)' \tag{S5}$$

and

$$-\mathscr{V}a' = D_a a'' - m\rho_c a\,, \tag{S6}$$

where prime denotes d/dz. From the numerical solution of the system with nonzero cell influx at the boundary, we find that the consumer density behind the leading front is constant "in the bulk", i.e. $\rho_c = \rho_c^\dagger = const.$. In the comoving frame, Eq.(S6) with constant $\rho_c^\dagger$ then allows us to make the ansatz $a \propto \exp(\lambda z)$ and obtain

$$-\mathscr{V}\lambda = D_a\lambda^2 - m\rho_c^\dagger\,. \tag{S7}$$

Using this ansatz we can write $a'/a = \lambda$, and integrate Eq.(S5) from $z^\dagger$ inside the bulk to $z = \infty$, subject to conditions $\rho_c(z^\dagger) = \rho_c^\dagger$, and $\rho_c(\infty) = \rho_c'(z^\dagger) = \rho_c'(\infty) = 0$, to get:

$$\lambda = \mathscr{V}/\chi_c\,. \tag{S8}$$

Note that the diffusion term vanishes due to the constant (i.e. "flat") bulk density. Using Eq.(S7) we thus obtain for the front speed

$$\mathscr{V} = \chi_c\sqrt{\frac{m\rho_c^\dagger}{D_a + \chi_c}}\,, \tag{S9}$$

or in the nondimensionalized system, leading to

$$\mathscr{V} = \tilde{\chi}_c\sqrt{\frac{\rho_c^\dagger}{1 + \tilde{\chi}_c}}\,. \tag{S10}$$

Interestingly, this result indicates that the Keller-Segel system can exhibit traveling wave solutions where the velocity is selected by the boundary influx, which fixes the bulk density $\rho_c^\dagger$. Therefore, the system does not

rely on the Kolmogorov criterion to have sharply localized initial cell densities to select a speed, in contrast with Fisher-KPP waves [12]. From a biological perspective, this can be a robust mechanism to facilitate wave-like migration through the interaction with a chemical field. A similar expression for the traveling wave speed had been found in a model of angiogenesis [13] in the absence of cell influx, where the cell density at the left boundary ($z = 0$) was assumed to be fixed. We also note that an expression for the front speed was recently obtained for bacterial chemotaxis with cell growth [9], which did not explicitly depend on the time scale of attractant consumption given by $(m\rho_c^\dagger)^{-1}$, but on the growth rate of bacteria in the bulk, highlighting how cell influx vs. growth might complementarily act to generate traveling waves.

S1.3 Chemoattractant kinetics in closed vs. open systems

Time evolution of the chemoattractant density as governed by Eq.(S2) indicates that at steady state, attractant concentration becomes $a^{\mathrm{st}} = 0$ as there is no external supply to balance its consumption by the chemotactic cell population. With respect to chemoattractant kinetics, this choice thus corresponds to a *closed* system with no in- or outflux of the attractant between the bulk of the system and the exterior. Experimentally, migration assays in a microfluidic channel with two holes at the ends approximate such a closed system, as after equilibration there are no external reservoirs to further supply chemoattractants into the migration channel. Alternatively, we can envisage an *open* system, where attractant molecules can enter or exit the system through the boundaries, leading to an effective turnover kinetics in addition to the consumption by cells. This choice thus corresponds experimentally to under-agarose migration assays, as the confined cell migration zone is in constant contact with a large reservoir of chemoattractant [14]. More generally, any setup where the cell migration zone is surrounded by semi-permeable boundaries that only allow the chemoattractant molecules to be transferred would be described by such turnover kinetics. In the context of immune cell migration in vivo, for instance, these two choices describe two limits where the chemoattractant molecules are either in fixed amount or constantly replenished along the path of migration.

In addition to the internalization by consumer-sensor cells, time evolution of the chemoattractant concentration in an open system then becomes:

$$\partial_t a = D_a \nabla^2 a + \underbrace{r - ka}_{\text{turnover}} - \underbrace{m\rho_c a}_{\text{uptake by cells}} , \tag{S11}$$

where r is a target concentration rate that describes the attractant influx into the system, and k is an effective "decay" rate for the outflux or loss of chemoattractants.

Nondimensionalization. Depending on the choice of closed vs. open systems, i.e. in the absence vs. presence of a chemoattractant turnover term in Eq.(S11), we obtain different spatiotemporal rescaling factors for nondimensionalizing the coupled PDE system. For the open case with nonzero turnover in Eq.(S11), we can rescale the cell and attractant concentrations to reduce the independent parameters r and k. Introducing the

transformations $t \to k^{-1}t'$, $x \to \sqrt{\frac{D_a}{k}}x'$, $\rho_c \to \frac{k}{m}\rho_c'$ and $a \to \frac{r}{k}a'$, and after dropping the primes we obtain:

$$\partial_t \rho_i = \tilde{D}_i \nabla^2 \rho_i - \tilde{\chi}_i \nabla \cdot \left(\rho_i \frac{\nabla a}{a} \right), \tag{S12}$$

and

$$\partial_t a = \nabla^2 a + 1 - a - \rho_c a, \tag{S13}$$

where again $\tilde{D}_i \equiv D_i/D_a$ and $\tilde{\chi}_i \equiv \chi_i/D_a$ are the reduced control parameters, as in the closed case. Note that, even though we have the two additional parameters r and k for the chemoattractant turnover, the nondimensionalized form of the open system is still controlled by the four rescaled parameters $\tilde{D}_i$ and $\tilde{\chi}_i$.

Argument for non-existence of traveling waves with turnover. Here we briefly discuss a possible physical argument on the non-existence of traveling wave solutions in the open system with attractant turnover. When including turnover kinetics $r - ka$, see Eq.(S11), the attractant profile relaxes toward a uniform background concentration $a(t \to \infty) = r/k$. This introduces a global restoring force that opposes the formation of sustained, cell-induced gradients in the attractant field. Mathematically, if we consider the same traveling wave ansatz used for the closed system with $a \propto \exp(\lambda z)$, we see that this cannot hold for all z due to the presence of the constant source term r, which is incompatible with an exponential decay. In other words, the ansatz fails, indicating that a self-sustained traveling front shaped by cellular uptake and chemotactic response cannot be maintained in the presence of attractant turnover. This highlights how external regulation of the chemical field can directly modulate self-organized wave propagation.

S2 Model predictions and sensitivity analysis

S2.1 Effects of parameter variations and temporal evolution

Here, we explore how different choices of control parameters affect model behavior, and examine the transient dynamics leading to long-term migration patterns.

Single-population dynamics. The simplest example for chemotaxis via self-generated gradients can be explored by looking at the dynamics of a single consumer cell type. The nondimensional system of equations then indicate that the consumer migration is controlled by the rescaled diffusion and chemotactic coefficients $\tilde{D}_c$ and $\tilde{\chi}_c$, respectively. We find in particular that the ratio $\tilde{\chi}_c/\tilde{D}_c$ (or χ_c/D_c) is the key control parameter that describes the transition of consumers from exhibiting diffusive-like to chemotactic migration profiles. Indeed, for $\chi_c/D_c > 0.4$, we found that the spatial profiles of consumers show a well-defined density peak, see Fig.S1A. To define such a peak, we use the convention that the cell density at the boundary should not be larger than half of the maximal cell density, i.e. $\rho(x=0) < \rho_{max}/2$. Furthermore, in this chemotactic regime, the long-time scaling of the mean position $\langle x \rangle \propto t^\alpha$ had exponents much larger than for simple diffusion, with typically $\alpha > 0.65$. In contrast, for $\chi_c/D_c < 0.4$ density peaks were not as pronounced and generically exhibited $\rho_{max}/2 < \rho(x=0)$,

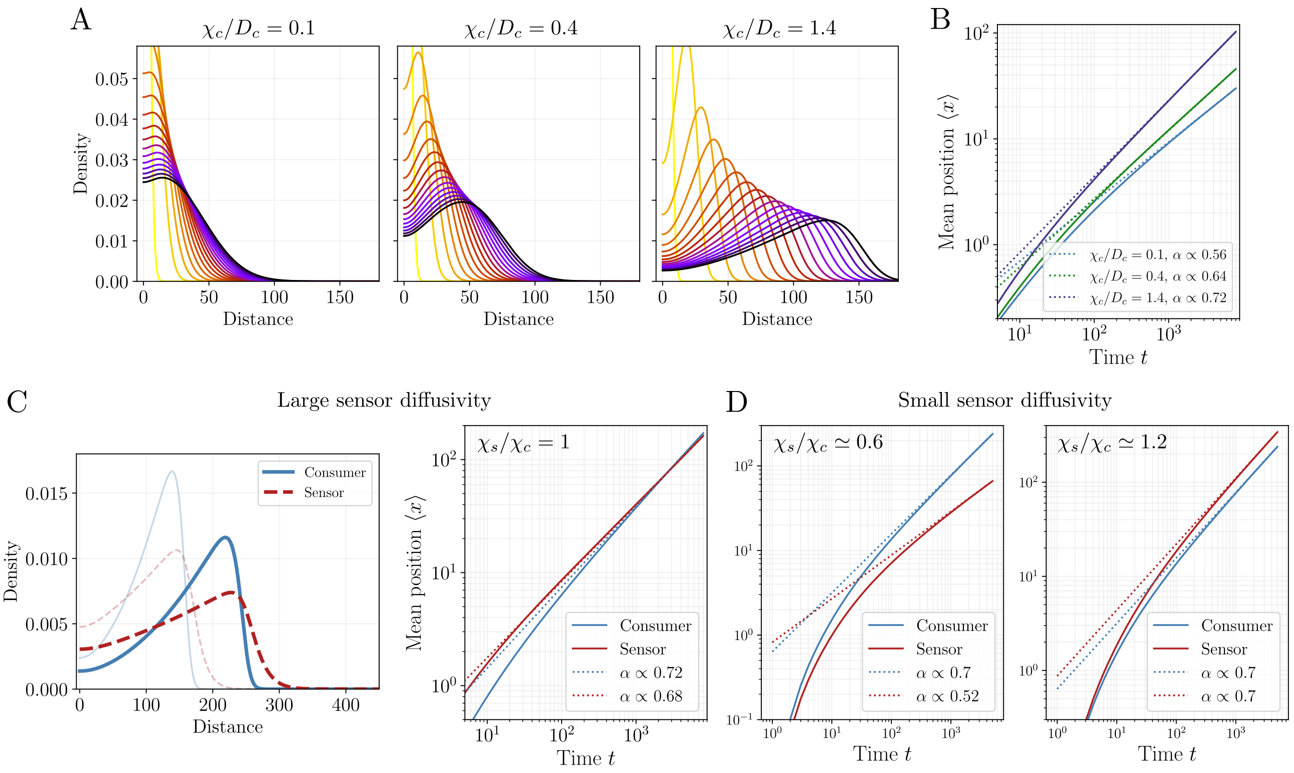

Supplementary Figure S1: **Model predictions for different parameter regimes.** A-B) Migration dynamics for a single consumer cell population. A) Spatial cell density profiles over time (time points color-coded) for three different choices of consumer chemotactic strength χ_c/D_c. For $\chi_c/D_c \leq 0.4$, the cell density on the left boundary remains relatively high, exceeding the half-peak density $\rho_{max}/2$ (left and middle panels), in contrast with the strongly chemotactic case with $\chi_c/D_c = 1.4$, where a well-defined density peak can be observed (right panel). B) Mean position of the cell population as a function of time ($\langle x \rangle \propto t^{\alpha}$) shows larger scaling exponents α with increasing χ_c/D_c. C-D) Influence of sensor diffusion coefficient D_s on the migration patterns. C) Cell density profiles (left) of the consumer and sensor population for identical chemotactic coefficients $\chi_c = \chi_s$ but with a large rescaled diffusion coefficient for the sensor population with $D_s/D_c = 5$. Long-time scaling of the mean position (right) shows that the sensor population has a smaller exponent $\alpha_s < \alpha_c$ and eventually falls behind the consumer cell population. D) Mean position scaling for the case when the sensor population has a smaller diffusion coeffcient than that of the consumers with $D_s/D_c = 0.083$. For $\chi_s/\chi_c \simeq 0.6$ (left), i.e. in the uncoupled regime, the sensor population falls behind the consumer cells with $\alpha_s < \alpha_c$. For $\chi_s/\chi_c \simeq 1.2$, i.e. in the coupled regime, sensors can propagate ahead of the consumer cell popoulation where the scaling exponents match with $\alpha_s = \alpha_c \simeq 0.7$.

while the scaling exponent decayed to $\alpha \leq 0.65$ and converged to $\alpha = 0.5$ for $\chi_c/D_c \to 0$, e.g. $\alpha \simeq 0.55$ for $\chi_c/D_c = 0.1$, see Fig.S1B.

Variations in the diffusion coefficients. Including the second population, i.e. sensor cells, in the system, we first wanted to test the influence of variations in their random motility as controlled by the rescaled diffusion coefficient $\tilde{D}_s$. Setting $\chi_c/D_c = 3$ for sufficiently chemotactic consumer cells, and identical chemotactic coefficients for consumers and sensors e.g. $\tilde{\chi}_s = \tilde{\chi}_c$, we found that a large diffusion coefficient for sensors (e.g. $D_s/D_c = 5$) led to slowly spreading densities for the sensor cell population, with a scaling exponent of $\alpha_s < \alpha_c$, which resulted in sensor cells falling behind the consumer population at long times (see Fig.S1C). Furthermore, we found that decreasing the diffusion coefficient of the sensor cells to be smaller than that of the consumers ($D_s/D_c < 1$) did not influence the coupled vs. uncoupled regimes, where $\chi_s/\chi_c < 1$ led to mean position ratios

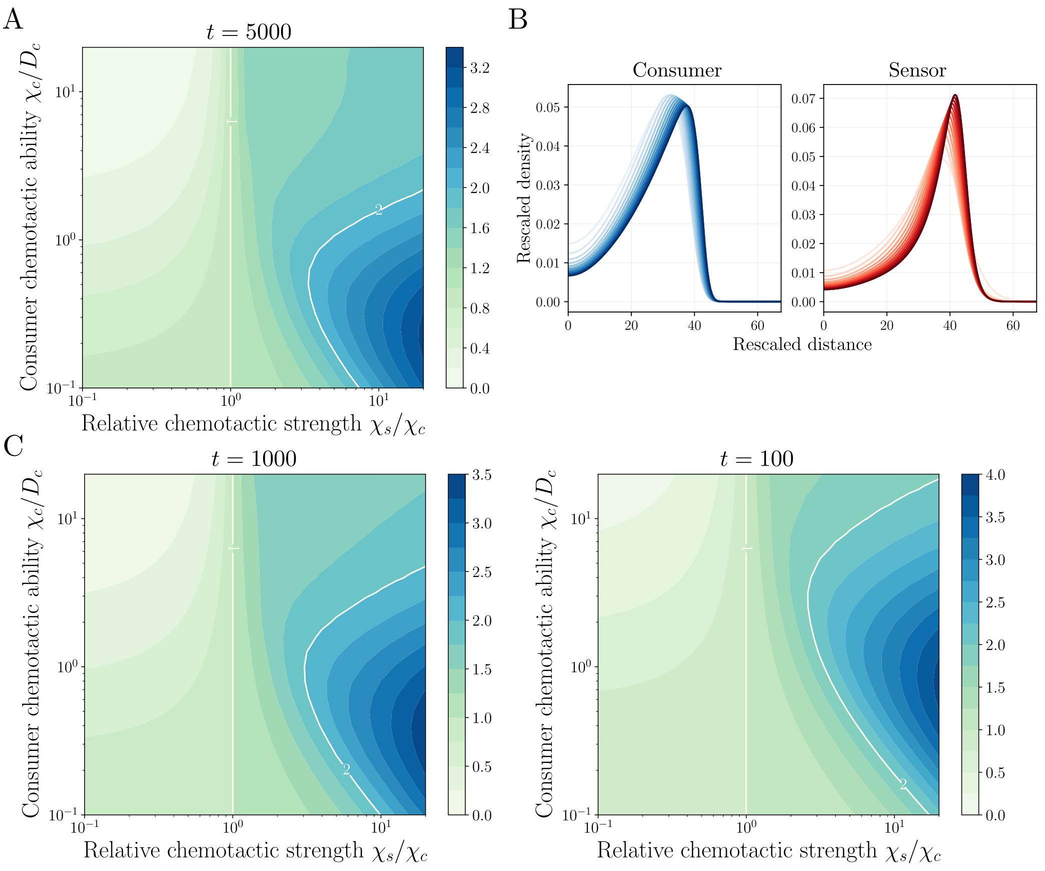

Supplementary Figure S2: **Temporal dynamics of the migration patterns.** A) Phase diagram of mean position ratios $\bar{x} \equiv \langle x_s \rangle / \langle x_c \rangle$ at a late time point ($t = 5000$) for the case of identical rescaled diffusion coefficients for sensor and consumer cell populations with $\tilde{D}_s = \tilde{D}_c = 0.1$. In contrast with the phase diagram shown in the main text (where $\tilde{D}_s \neq \tilde{D}_c$), for identical diffusion coefficients the mean position ratio fulfils $\bar{x} < 1$ in the entire uncoupled regime with $\chi_s/\chi_c < 1$. The remaining features of the phase diagram are largely preserved, in particular the bounded increase of $\bar{x}$ for sufficiently chemotactic consumer cells with a large chemotactic ability χ_c/D_c. B) Density profiles of both consumer (left) and sensor (right) populations exhibit an approximately scale-invariant form over distinct times (shaded colors) using transformations $x \to x(t^{\varphi})$ and $\rho_i \to \rho_i(t^{\theta})$ with appropriate rescaling exponents φ and θ. C) Phase diagrams of mean position ratios $\bar{x}$ at early time points of $t = 1000$ (left) and $t = 100$ (right) exhibit qualitatively similar features as in the long-time limit, with most changes observed at early times for large χ_s/χ_c.

of $\bar{x} \equiv \langle x_s \rangle / \langle x_c \rangle < 1$ with scaling exponents $\alpha_s < \alpha_c$ in the uncoupled regime, and for $\chi_s/\chi_c \geq 1$ we always recovered $\bar{x} > 1$ with $\alpha_s \simeq \alpha_c$ indicating the long-time coupling (see Fig.S1D). Finally, for identical consumer and sensor diffusibilities ($\tilde{D}_s = \tilde{D}_c$) the phase diagram of mean position ratios exhibited $\bar{x} < 1$ in the entire uncoupled region ($\chi_s/\chi_c < 1$) for all choices of consumer chemotactic ability χ_c/D_c (see Fig.S2A).

Slow-time dynamics of the system. Even though the migration dynamics in the absence of cell influx necessarily consists of slowly decaying density profiles, i.e. transient migration patterns, we found that the phase diagram as well as the scaling dynamics were preserved over long times, see Fig.S2C for a comparison of the mean position ratio phase diagram at different time points. Indeed, we found that the density profiles could be approximately mapped onto a scale-invariant form with appropriate rescaling factors, see Fig.S2B. This suggests that the diffusive leakage of cells from the propagating front does not influence the coupling mechanism over long times.

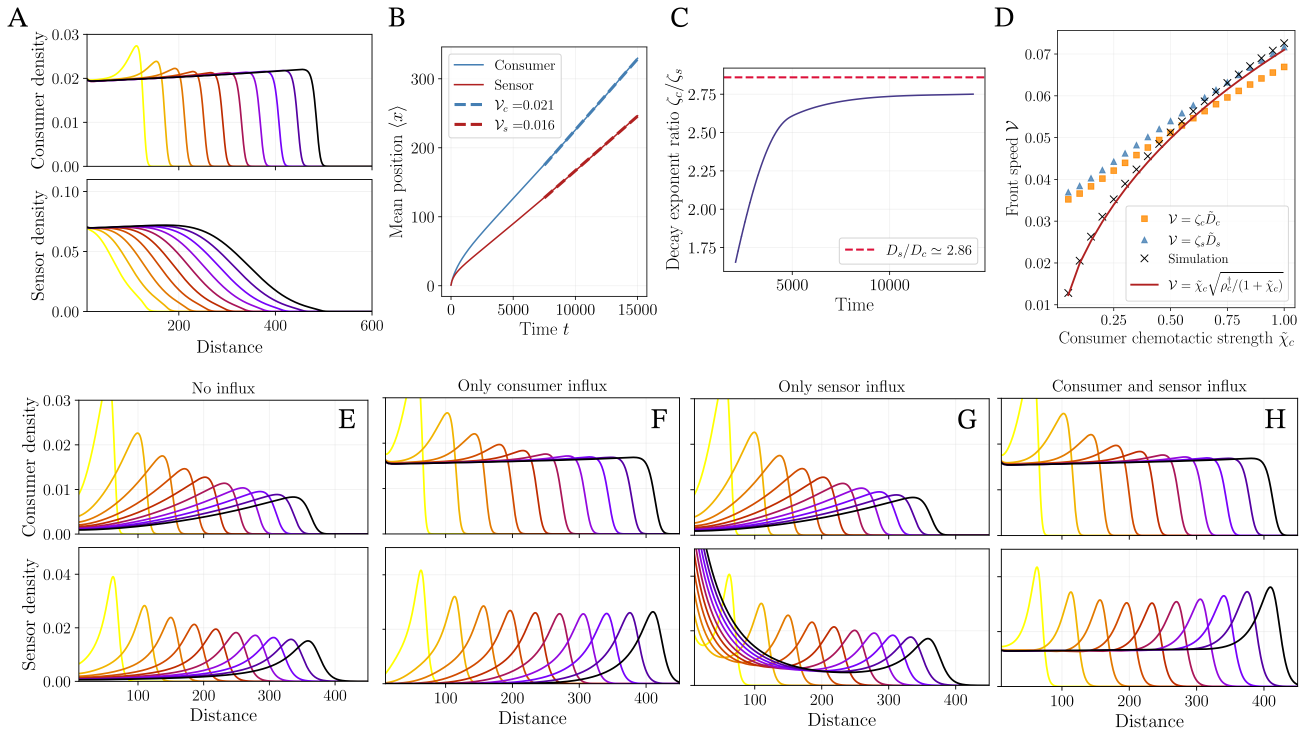

Supplementary Figure S3: **Dependence of traveling wave solutions and front speed on different parameter choices in the closed system.** A) Traveling wave profiles for different time points (color coded) in the case of an uncoupled migration pattern with $\chi_s < \chi_c$, where sensor cells lag behind the propagating consumer cell front (with $\tilde{\chi}_c = 0.3$ and $\tilde{\chi}_s = 0.24$). B) Mean position of cell densities over time indicate that the velocity-coupling breaks down for $\chi_s < \chi_c$. The consumer cell population propagates with a larger velocity ahead of the sensor cell population. C) Numerical test for estimating the diffusion coefficient ratio of the two cell populations D_s/D_c from their decay profiles given by the relation $\rho_i \propto \exp(-\zeta_i z)$ with $\zeta_i = \mathscr{V}/\tilde{D}_i$. Decay exponents fitted for the two cell populations at every time point exhibit a ratio ζ_c/ζ_s over time that approaches the diffusion coefficient ratio D_s/D_c. D) Numerical test for the analytical prediction of the traveling wave speed $\mathscr{V}$ (given by Eq.(S10)) for different choices of the consumer chemotactic coefficient $\tilde{\chi}_c$. Analytical estimate (red line) very closely approximates the front speed obtained from the numerical solution of the PDEs (crosses). In comparison, velocity estimates inferred from the decay profiles of cell densities by the relation $\mathscr{V} = \zeta_i \tilde{D}_i$ (squre and triangular markers) fail to describe the numerical wave speed for small $\tilde{\chi}_c$. E-H) Spatiotemporal profiles for cell densities for different influx conditions at the left boundary: no influx of either consumer or sensor cells (E), only consumer cells are added (F), only sensor cells are added (G), and both consumer and sensor cells are added (H). Only for the cases with a nonzero consumer cell influx (F and H) traveling waves can be formed. Sensor cell influx alone (G) cannot drive their wave-like migration pattern but instead leads to a slowly decaying density profile as in the case of no boundary influx (E). Parameter choices for (E-H) are $\tilde{\chi}_c = 0.2$, $\tilde{\chi}_s = 0.24$, $\tilde{D}_c = 0.07$, $\tilde{D}_s = 0.2$ and influx rate $\varepsilon = 0.001$ for both cell types.

S2.2 Influence of model assumptions on traveling wave properties

Uncoupled regime with cell influx. We next asked whether the predictions for the uncoupled regime with $\chi_s/\chi_c < 1$, where sensor cells fell behind the consumer cell population (as shown in Fig.1 in the main text), also held true for the system with nonzero influx of cells at the left boundary. We used the same diffusion coefficients for consumers and sensors as in the main text and only changed the sensor chemotactic coefficient to be slightly smaller than the consumer chemotactic strength with $\tilde{\chi}_s = 0.24$ and $\tilde{\chi}_c = 0.3$. This already led to a markedly diffferent migration pattern for the sensors: Spatial profiles did not exhibit any density peaks at any time point and the majority of the sensor density remained in the back of the consumer front, see Fig.S3A. Furthermore, the mean position of cell densities over time indicated that the velocity coupling observed in the coupled regime

disappeared in the uncoupled regime: Sensor cells propagated with a smaller velocity than the consumer cell population over long times, see Fig.S3B.

Spatial decay exponents of the density profiles. As we observed traveling waves for the case of cell influx through the boundary, we could switch to a comoving frame as a standard method to analyze stationary features [15], and arrive at the simple expression for the cell density given by Eq.(S5). At the leading tail of the traveling front, i.e. for saturated flat regions of the chemoattractant concentration with $a' \simeq 0$, this equation then dictates that the density profiles should scale as

$$\rho_i \propto \exp(-\zeta_i z) = \exp(-\mathcal{V} z/\tilde{D}_i)\,, \tag{S14}$$

where the subscript $i = c, s$ denotes the consumer or sensor population. This means that both cell densities will have exponential tails with decay lengths given by $\zeta_i \propto \tilde{D}_i^{-1}$. We can thus use this information to directly read off the relative diffusion coefficients from the density profiles of the cell populations using $\zeta_c/\zeta_s = D_s/D_c$. To test this prediction, we first looked at the scaling of cell density profiles around sufficiently flat regions of the chemoattractant concentration, i.e. for large z values. We first confirmed that density profiles indeed exhibited long exponential tails at saturating regions of the attractant gradient (i.e. ahead of the propagating fronts), as qualitatively predicted from the theory. After determining the decay exponents ζ_i from exponential fits to the density profiles, we then calculated the ratio ζ_c/ζ_s to see if this reproduced the ratio of diffusion coefficients used as input. From the numerical solution for the time evolution of density profiles we observed that the decay exponent ratio indeed approached D_s/D_c, see Fig.S3C.

Numerical test of the predicted traveling wave velocity. A concrete prediction from the analytical result for the front velocity, as given in Eq.(S10), is that it should scale with the square-root of the consumer cell chemotactic coefficient, i.e. $\mathcal{V} \propto \sqrt{\tilde{\chi}_c}$. To test this prediction, we systematically varied the rescaled chemotactic coefficient $\tilde{\chi}_c$ of the consumer cells and numerically calculated the front speed. We then compared the numerical values with the analytical prediction given by Eq.(S10), where we determined the bulk consumer density $\rho_c^\dagger$ from the final shape of the numerical density profiles. We found that the analytical prediction very closely matched the numerical values for the front speed for the entire range of $\tilde{\chi}_c$ values, see Fig.S3D. We then checked to what degree the approximate relation $\mathcal{V} = \zeta_i D_i$, see Eq.(S14), which strictly applies only for small chemoattractant gradients ($a' \simeq 0$), held in this range of parameters. After determining the decay exponents ζ_i from exponential fits to the spatial density profiles, we then compared this estimate with the numerical and analytical values for the front speed, and found that it only provided a good approximation for sufficiently large chemotactic coefficients $\tilde{\chi}_c$ but failed to reproduce the correct speeds otherwise, see Fig.S3D.

Boundary influx of only consumer or sensor cells. Although in the main text (see Fig.3) we discuss the case where there is a nonzero boundary influx of both consumer and sensor cells into the system, the choice of which cell type is added to the system might in general change the migration dynamics. To address this, we

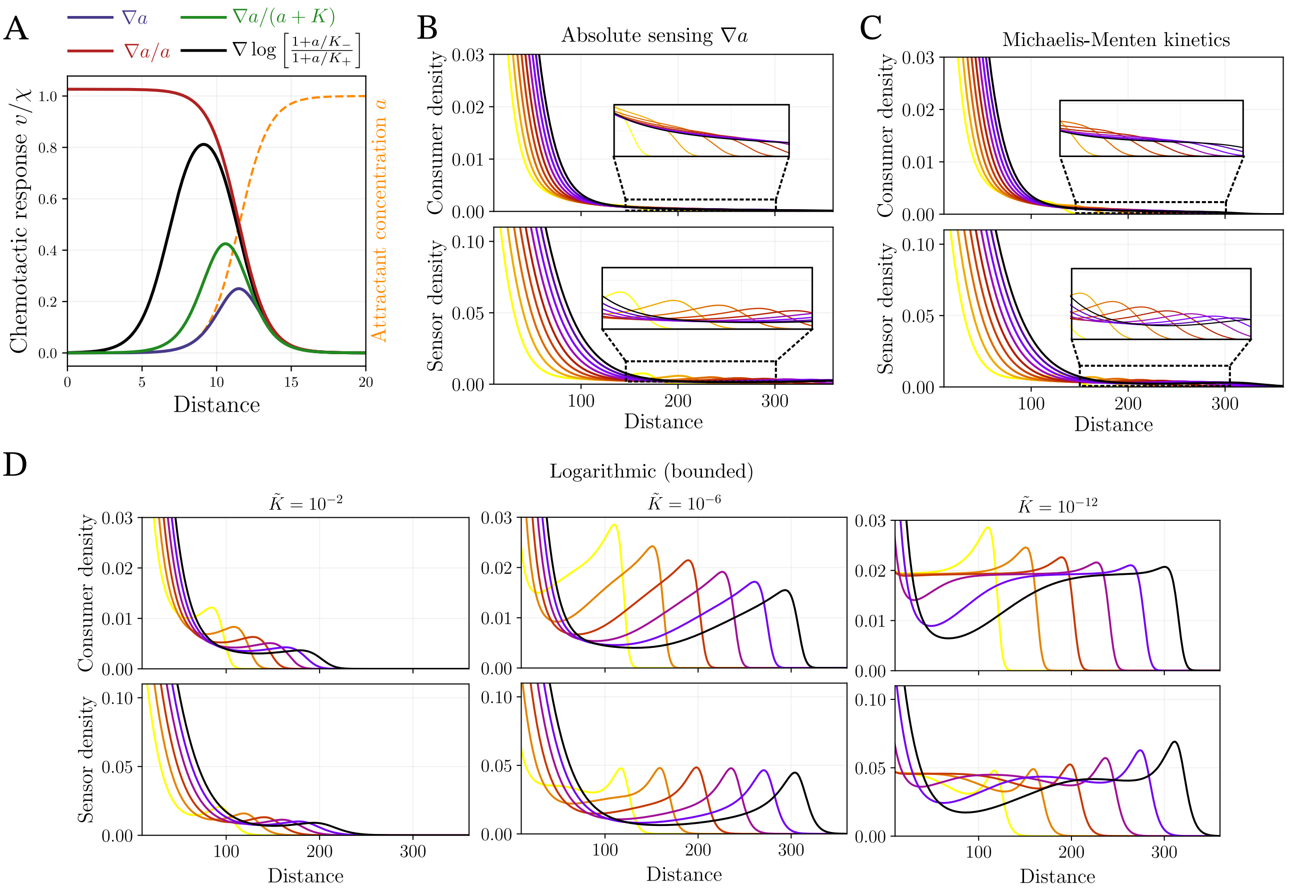

Supplementary Figure S4: **Dependence of traveling wave solutions on different chemotactic response functions in the closed system.** A) Illustration of different chemotactic response functions $\chi(a)$. For an attractant profile with an exponential tail (dashed line), absolute (purple) and relative (red) gradient sensing, Michaelis-Menten kinetics with half-maximum concentration K (green), and bounded logarithmic sensing (black) within upper and lower ranges K_+ and K_- are shown. Bounded logarithmic and Michaelis-Menten sensing functions can be tuned to collapse onto the relative sensing form for small $\tilde{K} \equiv K_-/K_+$ and small K, respectively. B-D) Time evolution of density profiles for the cases of absolute sensing (B), Michaelis-Menten kinetics (C), and bounded logarithmic sensing (D). In the latter case, for sufficiently small $\tilde{K}$ we start to observe transient density peaks, where cells in the bulk are partially recruited to the leading front (left and middle panels). Only for very small values $\tilde{K} \simeq 10^{-12}$, traveling wave-like density profiles can be obtained (right panel), consistent with the fact that in the limit $\tilde{K} \to 0$ bounded logarithmic sensing converges to relative sensing. Parameter choices for the different cases are: $\tilde{\chi}_c = 3$, $\tilde{\chi}_s = 3.6$ for (B) and (C); $\tilde{\chi}_c = 0.3$, $\tilde{\chi}_s = 0.36$ and $\tilde{K}$ as indicated in the plots for (D), and the rescaled diffusion coefficients are $\tilde{D}_c = 0.07$ and $\tilde{D}_s = 0.2$ for all cases.

briefly explored two additional cases: influx of only consumer cells and influx of only sensor cells. We found that only consumer cell influx is sufficient to generate traveling waves for *both* populations, see Fig.S3E-H. In contrast, sensor-only influx leads to a diffusive "leakage" of sensor cells into the back of the wave and fails to produce a coherent traveling wave pattern, despite their constant supply. These results thus further support the notion that the migration pattern of sensors is tightly coupled to the presence of consumer cells, and that active gradient-shaping by consumers drives the wave formation.

Influence of different chemotactic response functions on the traveling wave pattern. We next turned to test the robustness of the traveling wave solutions to different types of chemotactic response functions, as the cells' sensing ability might be constrained by the absolute concentration of the chemoattractant. In the

minimal logarithmic response defined by $\mathbf{v} = \chi \nabla \log(a)$, for instance, as $a \to 0$ the cells would still have a constant response, e.g. for an exponentially decaying attractant profile. Therefore, we now briefly discuss three additional response functions given by (i) absolute gradient sensing $\mathbf{v} = \chi \nabla a$, (ii) sensing dictated by Michaelis-Menten kinetics $\mathbf{v} = \chi \nabla a/(a+K)$ with half-maximum concentration K, and (iii) logarithmic sensing $\mathbf{v} = \chi \nabla \log[(1+a/K_-)/(1+a/K_+)]$ bounded within lower and upper concentration limits K_- and K_+, respectively. Fig.S4A illustrates the different response functions for an attractant profile with an exponentially decaying tail given by $a \propto \exp(x)/(C+\exp(x))$, where C is a sufficiently large constant.

We then asked whether these different forms of chemotactic response functions had an influence on the migration patterns, in particular with respect to the existence and dynamics of traveling wave profiles. To explore these cases, we first nondimensionalized the coupled PDEs for each choice of chemotactic sensing function. This could be done by introducing a rescaling for the attractant concentration as $a \to Ka'$ for the Michaelis-Menten kinetics, and as $a \to K_+ a'$ for bounded logarithmic sensing. The corresponding nondimensional equations for the cell density evolution then followed $\partial_t \rho_i = \tilde{D}_i \nabla^2 \rho_i - \tilde{\chi}_i \nabla \cdot (\rho_i \nabla a/(a+1))$ and $\partial_t \rho_i = \tilde{D}_i \nabla^2 \rho_i - \tilde{\chi}_i \nabla \cdot (\rho_i \nabla \log[(1+a/\tilde{K})/(1+a)])$, respectively. The latter equation for logarithmic sensing thus introduces a new rescaled parameter $\tilde{K} = K_-/K_+$ that is given by the ratio of the lower and upper concentrations.

Numerical solutions of the PDEs showed that deviations from the relative sensing led to notable changes in the cell density evolution: First of all, we did not observe traveling wave solutions for any of the alternative sensing mechanisms. Next, both absolute sensing and Michaelis-Menten kinetics resulted in accumulation of cells close to the boundary of the system, and even for the case of highly chemotactic cell populations with $\tilde{\chi}_c/\tilde{D}_c \simeq 40$, consumer cells did not form well-defined density peaks, see Fig.S3B-C (insets). In contrast, bounded logarithmic sensing with a small $\tilde{K} \equiv K_-/K_+$ led to transient peaks of consumer cells that decayed slowly in time, see left and middle panels of Fig.S4D, indicating that in this case cells in the bulk could be partially recruited to the leading front. For very small values $\tilde{K} \leq 10^{-12}$ we found that traveling wave-like density patterns also emerged with bounded logarithmic sensing, see right panel in Fig.S4D, as expected from the convergence of this sensing type to the relative sensing in the limit of small $\tilde{K}$.

S3 Parameter estimates, model fitting and perturbation experiments

In this section we briefly outline the inference of key parameters of the system, quantitative inferences for the modelling assumptions from experimental data, perform further parameter scans for testing model robustness, and describe perturbation experiments performed to test the predictions of the theory. Parameter estimates that are used for the comparison with experimental data are summarized in Table S1.

Parameter	Estimate
Rescaled consumer diffusion coefficient $\tilde{D}_c$	0.07
Rescaled sensor diffusion coefficient $\tilde{D}_s$	0.2
Rescaled consumer chemotactic coefficient $\tilde{\chi}_c$	$0.2^\dagger$, $1.5^\ddagger$
Rescaled sensor chemotactic coefficient $\tilde{\chi}_s$	$0.24^\dagger$, $1.8^\ddagger$
Cell influx rate ε	0.001
Temporal rescaling factor $\eta = (m\bar{\rho}_c)^{-1}$	0.1min†
Temporal rescaling factor k^{-1}	0.02min‡

Table S1: **Parameter values used in the numerical evaluation of the nondimensional system of equations.** Dagger symbols (†) indicate parameter values used for the comparison with the data from microfluidic channel experiments, while double-dagger symbols (‡) correspond to estimates for the comparison with the under-agarose experiments. Other parameter values used in the analysis of alternative model predictions are indicated in the corresponding figure captions.

S3.1 Inference of diffusion coefficients

As the nondimensionalized system is entirely controlled by the rescaled diffusion and chemotactic coefficients $\tilde{D} \equiv D/D_a$ and $\tilde{\chi} \equiv \chi/D_a$, where D_a denotes the diffusion coefficient of the attractant, estimating the diffusion coefficients of the consumer and sensor cell populations is a key step to constrain the analysis for the experimentally relevant migration regime. We approached this using complementary methods to obtain reproducible estimates for the diffusion coefficients.

S3.1.1 Diffusion coefficient of consumer/dendritic cells

To extract the diffusive behavior of dendritic cells (DCs) independently from their chemotactic response, we looked at the migration data of dendritic cells (DCs) in under-agarose experiments in the absence of the chemoattractant CCL19, as we published previously [14]. We then set out to estimate the diffusion coefficient D_c employing a two-stage strategy: first based on the velocity autocorrelation function (VACF), and then via Bayesian inference methods using the Furth's formula for persistent random walks [16] describing mean-squared displacements (MSD). This complementary strategy thus combined a direct summary statistics of cell motility (via VACF) with a robust probabilistic fit to MSD data.

We confined our analysis to trajectories that had reached $T = 120$ mins to focus on sufficiently processive cells, and analyzed tracks from 2 different experiments with $N = 168$ cells (see Fig.S5A for an exemplary plot of cell tracks).

(i) Velocity autocorrelation analysis. For each cell, we first calculated the instantaneous velocities between discrete time points as $\mathbf{v}(t_i) = (\mathbf{x}(t_{i+1}) - \mathbf{x}(t_i))/\tau$, with the time interval $\tau = 1$min. We then calculated the velocity dot products $C_v(\Delta t) \equiv \langle \mathbf{v}(t) \cdot \mathbf{v}(t + \Delta t) \rangle$ for increasing time lags Δt, where $\langle\rangle$ denotes averaging over all

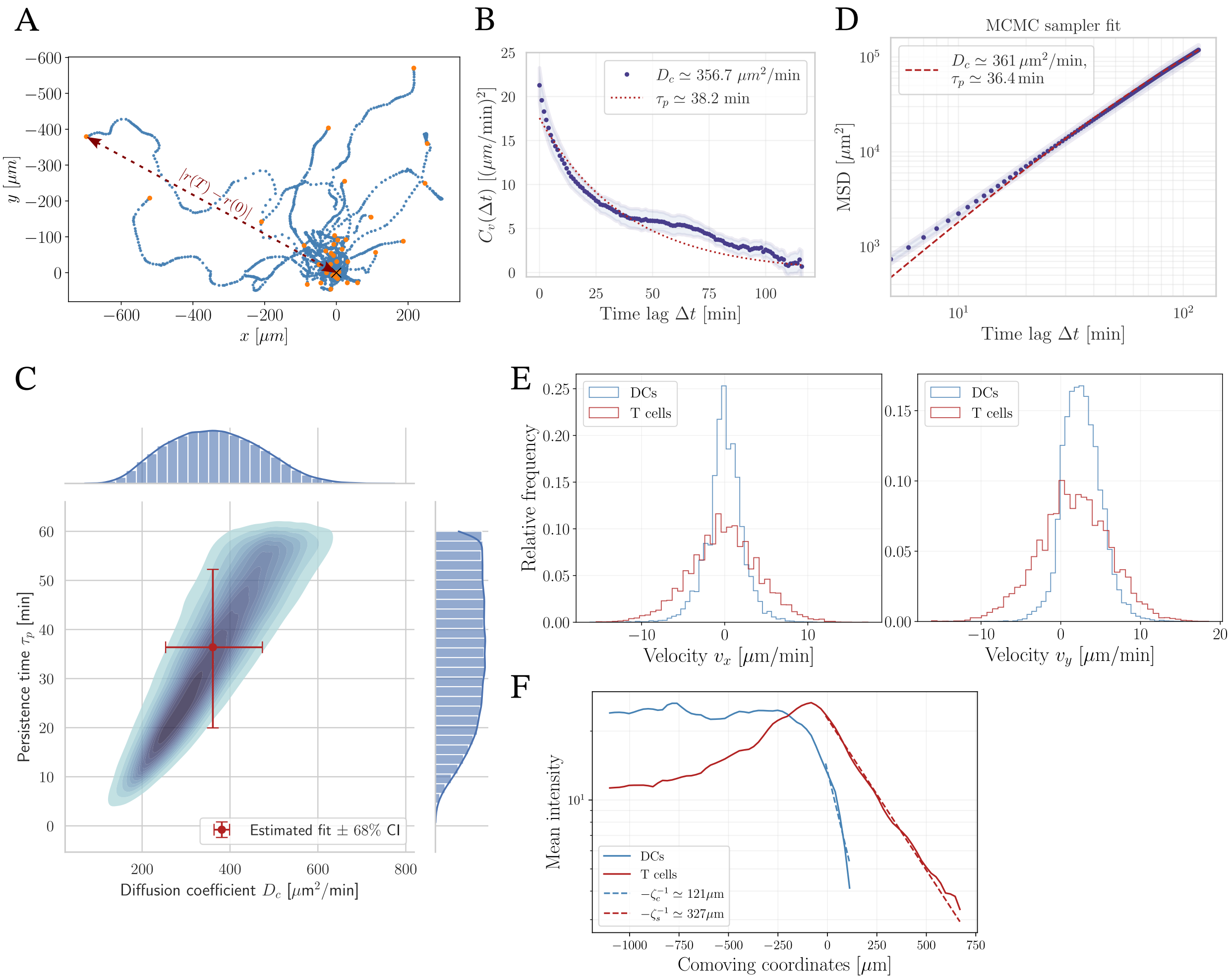

Supplementary Figure S5: **Estimation of the rescaled diffusion coefficients of DC (consumer) and T cell (sensor) populations.** A) Spider plot of exemplary DC trajectories from under-agarose experiments in the absence of the chemoattractant CCL19. For each cell trajectory, orange dots indicate the position of the corresponding cell at time point $T = 120$min, whereas blue dots describe its past coordinates. B) Average velocity autocorrelation function from $N = 168$ cell trajectories calculated using a "sliding window" for each time lag Δt. The estimated diffusion coefficient from the integral of the velocity correlation function is $D_c \simeq 357\mu m^2$/min. The exponential fit (red dotted line) to the average autocorrelation data indicates a persistence time of $\tau_p \simeq 28$ min. C) Joint *a posteriori* distributions of estimated DC diffusion coefficient D_c and persistence time τ_p obtained from MCMC sampling. The estimated fit values are shown with the red dot, together with 68% credible interval (error bars). The side histograms display the marginal distributions for D_c and τ_p. D) Calculation of the MSD by taking into consideration of each time point in a given cell trajectory. MSD is calculated using a "sliding window" for each time lag Δt. Average MSD datasets (circles) obtained from $N = 168$ cell trajectories are plotted over increasing time intervals and compared against a fit obtained from an MCMC sampler using Furth's formula (Eq.(S16)) as the *a priori* model (dashed red line). The MCMC estimates for $D_c \simeq 361\mu m^2$/min and $\tau_p \simeq 36$ min are very close to the values obtained from the velocity autocorrelation data in B). Error bars in (B,D) represent SEs. E) Histogram of dendritic (blue) and T cell (red) velocities obtained from the under-agarose assays in the presence of the chemoattractant CCL19. Both the $x-$ (left) and $y-$ (right) components of velocities show comparable fluctuations for each cell population, whereas T cell velocities have notably larger SDs than that of the DCs with $\sigma(v_s) \simeq 4.3\mu m/min$ vs. $\sigma(v_c) \simeq 2.3\mu m/min$, indicating a diffusion coefficient ratio of $D_s/D_c = \sigma(v_s)^2/\sigma(v_c)^2 \simeq 3.5$. F) Semi-log plot of the average density profiles of DCs (blue) and T cells (red) in the comoving frame of the DC front, obtained from the microfluidic channel experiments (profiles correspond to the one displayed in Fig.3D in the main text). Decaying tails of the profiles ($\rho_i \propto \exp(-\zeta_i z)$) are fitted by the length scales $\zeta_c^{-1} \simeq 121\mu$m and $\zeta_s^{-1} \simeq 327\mu$m, which indicate a diffusion coefficient ratio of $D_s/D_c \simeq 2.7$.

cells (see Fig.S5B). We fitted an exponential decay $C_v \propto \exp(-\Delta t/\tau_p)$ to estimate the persistence time τ_p, and

numerically integrated the VACF to calculate the diffusion coefficient using

$$nD_c = \int_0^T C_v(t)dt\,, \tag{S15}$$

where $T = 120$ min is the maximal time of cell trajectory data. From this analysis, we obtained approximate values $D_c \simeq 357\mu m^2/\text{min}$ and $\tau_p \simeq 38$ min.

(ii) Mean-squared displacement analysis and MCMC sampling. We then turned to a joint method of analyzing MSD data from cell trajectories by combining it with a Markov chain Monte Carlo (MCMC) sampler to obtain independent estimates for the diffusion coefficient together with robust error bars. We first determined the scaling of MSD of all trajectories (from $N = 168$ cells) over time (up to $T = 120$ mins and considering trajectories that were processive enough to exceed $\sim 50\mu$m radial distance). To infer both D_c and τ_p jointly from this dataset, we decided to use Furth's formula for persistent random walks [16]:

$$MSD(\Delta t) \equiv \langle (r(t+\Delta t) - r(t))^2 \rangle = 2nD_c(\Delta t - \tau_p(1 - e^{-\Delta t/\tau_p}))\,, \tag{S16}$$

where $n = 2$ is the dimension of the system and t denotes an arbitrary time point to define sliding averages over time.

We next implemented an MCMC sampler using the EMCEE package in Python [17]. To set this, we first defined a likelihood function based on Gaussian errors:

$$\mathscr{L} = \exp\left(-\tfrac{1}{2}\sum_i \left(\tfrac{MSD^*(\Delta t_i) - MSD(\Delta t_i)}{\sigma_i}\right)^2\right)\,, \tag{S17}$$

where MSD^* and MSD denote the experimentally observed data and the model prediction given by Eq.(S16), respectively, and σ_i is the estimated standard deviations of the MSD at time lag Δt_i . To validate the implementation of the MCMC sampler, we first used synthetically generated cell trajectories from an Ornstein-Uhlenbeck process with controlled diffusion coefficient D_c and persistence time τ_p by generating samples from velocity distributions with Gaussian errors $\sigma_v = D_c/\tau_p$ and decay times τ_p. The MCMC was initialized using a Gaussian spread (%20 relative variation) around a biased initial guess $D_c = 2D_c^{VACF}$ and $\tau_p = 1.2\tau_p^{VACF}$, where D_c^{VACF} and τ_p^{VACF} are the estimates obtained from the VACF analysis. Using uniformly distributed priors with $D_c \in [0, 3000]\mu\text{m}^2$/min and $\tau_p \in [0, 60]$min, we found that the MCMC setup accurately provided converging fit values that recovered different input diffusion and persistence parameters.

Having established the validity of the MCMC setup, we next proceeded to run it with 300 walkers and 5000 steps on the experimental MSD data with the same initialization settings. We found that the the posterior for D_c was well-defined and peaked very close to the independent VACF estimate, while the posterior for τ_p was broader and asymmetric, presumably reflecting the limited applicability of Furth's model to early-time MSD data, see Fig.S5C). The final parameter estimates from the posterior distributions were $D_c \simeq 361 \pm 100\mu\text{m}^2$/min, and $\tau_p \simeq 36 \pm 14$ min (median $\pm$ SD), which closely matched the values obtained from the VACF analysis and provided an excellent fit to the observed MSD curve (see dashed fit line in Fig.S5D).

Both the VACF-based and MCMC-based methods therefore indicated a consistent estimate for the diffusion coefficient of the DCs of around $D_c \simeq 360\mu m^2/\text{min}$, which was also consistent with the range reported previously for dendritic cells [18]. Using a chemoattractant diffusion coefficient of $D_a \simeq 86\mu m^2/s$ [14], we could then fix the rescaled diffusion coefficient of DCs by $\tilde{D}_c = D_c/D_a \simeq 0.07$.

S3.1.2 Diffusion coefficient of sensor / T cells.

To determine the diffusion coefficient of the T cells, we used experimental datasets from under-agarose experiments where DCs and T cells migrated in a uniform field of the chemoattractant CCL19, as published in [14]. As the T cell trajectories in this setup can potentially involve interactions with the DCs particularly in dense regions, we decided not to use the MSD or velocity autocorrelation data to estimate the T cell diffusion coefficient. Instead, we looked at the distributions of velocity components in the two (x & y) directions and compared their fluctuations both for the DC and T cell populations, see Fig.S5E for the velocity distributions. As the diffusion coefficient of the cells is proportional to the variance of velocity distributions, regardless of advective fluxes driven by the coupling to the chemoattractant, we could then infer the relative diffusion coefficient of the T cells (sensors) via:

$$D_s/D_c \propto \frac{\sigma(v_s)^2}{\sigma(v_c)^2} \equiv \frac{\langle (v_s - \langle v_s \rangle)^2 \rangle}{\langle (v_c - \langle v_c \rangle)^2 \rangle}. \tag{S18}$$

Interestingly, even though the mean velocities in the y direction reflect the bias due to advective chemotactic flux, we could separate the diffusive contribution as the variances in v_x and v_y exhibit similar values: The variance in v_x distributions were $\sigma(v_{s,x})^2 \simeq 17(\mu m/\text{min})^2$ for T cells and $\sigma(v_{c,x})^2 \simeq 5(\mu m/\text{min})^2$ for DCs, and the variance of the v_y distributions were $\sigma(v_{s,y})^2 \simeq 20(\mu m/\text{min})^2$ for T cells and $\sigma(v_{c,y})^2 \simeq 6(\mu m/\text{min})^2$ for DCs. This analysis indicated that the ratio between the diffusion coefficients of the sensor (T cell) and consumer (DC) populations was about $D_s/D_c \simeq 3.5$.

Furthermore, we considered another independent method to infer the ratio of diffusion coefficients for the case of traveling waves from the decay profiles of cell densities, as predicted from Eq.(S14) and numerically tested in Fig.S3C. Because traveling waves are obtained in the microfluidic channel experiments, we could then simply fit the decay lengths of the density profiles in the comoving frame with the traveling wave velocity $\mathscr{V}$. Using Gaussian filtering for the density profiles to better fit the exponential decay lengths, we found that the ratio of the decay lengths between the T cell (sensor) and DC (consumer) populations fell consistently within the range $\zeta_c/\zeta_s = D_s/D_c \simeq 2.7$ (see Fig.S5F), in good agreement with the diffusion ratio obtained from the variance of velocity distributions from the under-agarose experiments. These two independent methods from different experimental setups led us to conclude that the diffusion coefficient ratio of sensor and consumer populations is approximately given by $D_s/D_c \simeq 3$. Using the rescaled diffusion coefficient for consumer cells of $\tilde{D}_c = 0.07$, as estimated above, we then used the value $\tilde{D}_s = 0.2$ for the rescaled diffusion coefficient of the sensor cells.

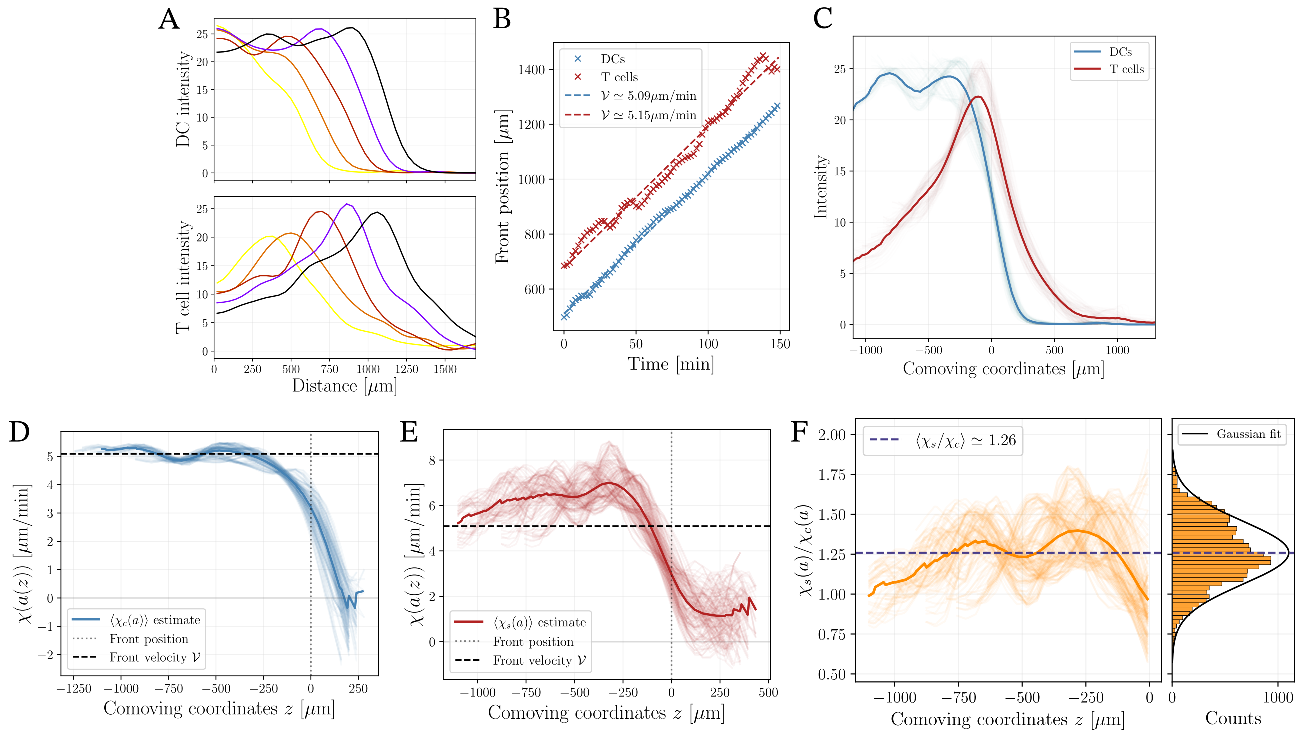

Supplementary Figure S6: **Estimation of chemotactic response functions and the relative chemotactic strength between cell populations.** A) Time evolution of DC (top panel) and T cell (bottom panel) profiles obtained from a Gaussian filtering of binned intensity data. The smoothened intensities are used to determine the position of the density front from the half-maximum of peak densities. B) Front positions of DC and T cell populations show that both cell populations propagate with the same constant speed $\mathcal{V} \simeq 5\mu$m/min and preserve the separation between their fronts over 150 mins. The velocities are estimated using least-squared linear fits. C) Smoothed intensity profiles of DCs (blue) and T cells (red) overlaid in the comoving frame. Shaded lines indicate intensity profiles at different time points and the thick lines indicate intensities averaged over time. D) Pointwise estimation of chemotactic response function $\chi_c(a(z))$ in the comoving frame $z = x - \mathcal{V}t$ obtained from Eq.(S21) using the DC intensities shown in C). Individual shaded lines represent estimates from different time points. For small z, $\chi_c(a)$ approaches the front speed $\mathcal{V}$ (dashed horizontal line). Average estimate $\langle \chi_c(a) \rangle$ (thick line) is approximately constant in the back of the wave and monotonically decays to zero around the DC front position, as expected from a relative chemotactic response function. E) Pointwise estimate of the chemotactic response function $\chi_s(a(z))$ obtained from the T cell intensity data. $\chi_s(a)$ estimates from different time points (shaded lines) show larger variations compared with those from the DC estimates in D), but generically obtain larger values. F) The ratio $\chi_s(a)/\chi_c(a)$ of pointwise estimates shown in D) and E) can be used to infer the relative chemotactic strength ratio χ_s/χ_c. The estimates of the ratio are obtained for individual time points (shaded lines) and and from the mean estimates (thick line) calculated at the rear of the wave $z \leq 0$ (left panel). Histogram of individual estimates pooled from each time point and at each spatial coordinate z can be fitted by a normal distribution with mean $\chi_s/\chi_c \simeq 1.26$ (dashed horizontal line) and standard deviation 0.2. Density profiles shown in (A) obtained from $n = 5$ individual experiments.

S3.2 Inference of chemotactic sensing functions and relative sensing strength χ_s/χ_c

Having obtained robust estimates for the diffusion coefficients of the cell populations, we next sought to infer their chemotactic response functions from experimental data. As the system is governed by dynamically evolving chemoattractant fields shaped by the propagating DC population, and direct observation of the attractant is not available, inference of the chemotactic response typically relies on different modelling assumptions and fitting procedures. However, we realized that in the presence of traveling waves, one can exploit the structure of the comoving reference frame $z = x - \mathcal{V}t$, using the experimentally observed wave speed $\mathcal{V}$.

Indeed, for a generic chemotactic cell population ρ, its dynamics in the comoving frame are governed by:

$$-\mathscr{V}\rho'(z) = D\rho''(z) - (\rho(z)\chi(a(z)))' \,, \tag{S19}$$

where $\chi(a(z))$ denotes the chemotactic response function (e.g. absolute/relative gradient sensing, bounded logartihmic sensing etc.), and primes denote differentiation with respect to z. Moreover, the conservation equation in the comoving frame implies a total flux balance given by:

$$J(z) = -D\rho'(z) + \chi(a(z))\rho(z) = \mathscr{V}\rho(z) \,, \tag{S20}$$

where the right-hand side reflects the mass transport associated with the wave. Rewriting this yields an expression for the chemotactic response function:

$$\chi(a(z)) = \frac{\mathscr{V}\rho(z) + D\rho'(z)}{\rho(z)} \,, \tag{S21}$$

i.e., it can be "read off" directly from the observed cell density profiles $\rho(z)$ in the comoving frame and the known wave velocity $\mathscr{V}$.

To apply this, we first performed Gaussian filtering on the time-resolved cell density profiles ρ_i to better estimate the front positions and the gradients $\rho_i'(z)$ (see intensity profiles in Fig.S6A). The front positions could now be reliably obtained by identifying the furthest spatial coordinate at which the intensity dropped to approximately half of the peak value. Using these front positions, we obtained a best fit for the front speed $\mathscr{V} \simeq 5\mu$m/min for both DC and T cell populations, see Fig.S6B. Using the inferred estimates for the diffusion coefficients D_i, we then calculated the response function $\chi(a(z))$ from the smoothed density data $\rho_i(z)$ in the comoving frame across different time points (Fig.S6C).

Averaging over all time points, we found that the chemotactic response function of the consumer (DC) population closely follows a relative sensing form, $\chi(a) \propto \chi \nabla a/a$, as this functional form remains approximately constant even at low attractant concentrations. This contrasts with absolute sensing, Michaelis-Menten sensing, or bounded logarithmic sensing, which decay more rapidly as $a \to 0$, see our discussion in Section S2 and Fig.S4. As expected for an exponentially decaying attractant concentration, $\chi_c(a)$ approaches constant values near the front speed $\mathscr{V}$ in the back of the wave at all time points, consistent with Eq.(S21) under approximately constant densities. For T cells, the inferred response function $\chi_s(a)$ was noisier, see Fig.S6E, but similarly did not decay significantly in the back of the wave and consistently showed larger values than $\chi_c(a)$ of the DCs, in line with the expectation that T cells respond more strongly to attractant gradients.

Because we do not have an independent method to estimate the gradient ∇a, we cannot infer the exact functional dependence of the chemotactic response. However, because the chemotactic coefficient χ_i appears as a prefactor to the response function $\chi_i(a)$ (regardless of its dependence on ∇a), we can directly estimate the *relative* chemotactic strength χ_s/χ_c by taking the ratio of the inferred functions $\chi_s(a)/\chi_c(a)$. This provides a direct, data-driven method to estimate the relative chemotactic coefficient between the sensor and consumer populations, which is a key parameter controlling the phase diagram of the system.

Calculating the ratio $\chi_s(a)/\chi_c(a)$ in the comoving frame across all time points yielded estimates predominantly above $\chi_s/\chi_c \geq 1$ in the back of the wave, see Fig.S6F (left panel). Aggregating these individual pointwise estimates into a joint histogram, we found that the distribution of χ_s/χ_c could be well fit by a normal distribution centered around $\chi_s/\chi_c \simeq 1.26$, with a standard deviation $\sigma_{\chi_s/\chi_c} \simeq 0.2$, see Fig.S6F (right panel).

S3.3 Parameter fitting and model robustness

After estimating the rescaled diffusion coefficients $\tilde{D}_i$ and the relative chemotactic strength χ_s/χ_c between sensor and consumer populations, the dynamics of the nondimensional system of equations is entirely controlled by the rescaled chemotactic coefficient $\tilde{\chi}_c$ of consumers together with the choice of initial and boundary conditions. As stated in the main text, we first numerically observed a traveling wave solution when there is a nonzero boundary flux of cells at $x = 0$. We used a small influx rate $\varepsilon = 0.001$ for comparison with both experimental setups, although this rate can be changed if a more quantitative match between absolute density values is needed. As we did not have quantitative data on the exact densities from the microfluidic channel experiments, but used relative intensity datasets instead, we did not tune this influx rate further.

Unlike the relative chemotactic coefficient ratio between sensor and consumers, which we determined to be around $\chi_s/\chi_c \simeq 1.2$ from experimental data, the choice for the rescaled chemotactic coefficient of the consumer cell population was relatively unconstrained, as any sufficiently large value with $\chi_c/D_c > 2$ led to traveling waves in the closed system. We therefore used $\chi_c/D_c \simeq 3$ to obtain a well-defined wave. In contrast, for the open system with attractant turnover, we chose $\chi_c/D_c \simeq 20$ to reproduce the experimental density profiles, although the relative chemotactic strength of the two cell populations was chosen to be $\chi_s/\chi_c \simeq 1.2$, i.e. identical to the closed system case. The initial conditions for the attractant profile in both systems were fixed by a constant spatial density with $a(x,t=0) = 1$. For the consumer and sensor cell populations we used sharply localized initial density profiles with exponentially decaying tails described by $\rho_i(x,t=0) = 1/(1+A\exp(x-B))$ with $A = B = 1$ for the closed system, and $A = 1$ and $B = 5$ for the open system.

Finally, to transform the nondimensional solutions to physical spatiotemporal units, we had to fix the rescaling factors in $t \to \eta t'$ for the closed system and in $t \to k^{-1}t'$ for the open system. To reproduce the experimentally observed migration dynamics we then set $\eta = 0.1$min and $k^{-1} = 0.02$min, which then also rescaled the spatial dimensions via $x \to \sqrt{D_a\eta}x'$ and $x \to \sqrt{D_a k^{-1}}x'$ for the closed and open systems, respectively. Table S1 summarizes the parameter estimates used for the comparison between numerical solutions and experiments.

We next tested the robustness of model predictions against variations in inferred parameter estimates from data. From the MCMC estimation of the diffusion coefficient D_c of consumers (DCs), see Fig.S5C, we obtained 95% credible intervals of $D_c \in [180-570]\mu$m^2/min. As the independent estimates for the diffusion coefficient ratio between the sensor (T cell) and consumer (DC) populations obtained from velocity fluctuation analysis and density profile decay lengths was $D_s/D_c \simeq 3.5$ and $D_s/D_c \simeq 2.7$, respectively, we used a non-conservative range for the error in this ratio of $D_s/D_c \in [2,4]$. We therefore used variations in diffusion coefficients (i) uniformly chosen from $D_c \in [180-570]\mu$m^2/min for consumer cells, and (ii) scaled diffusion coefficients for the sensors

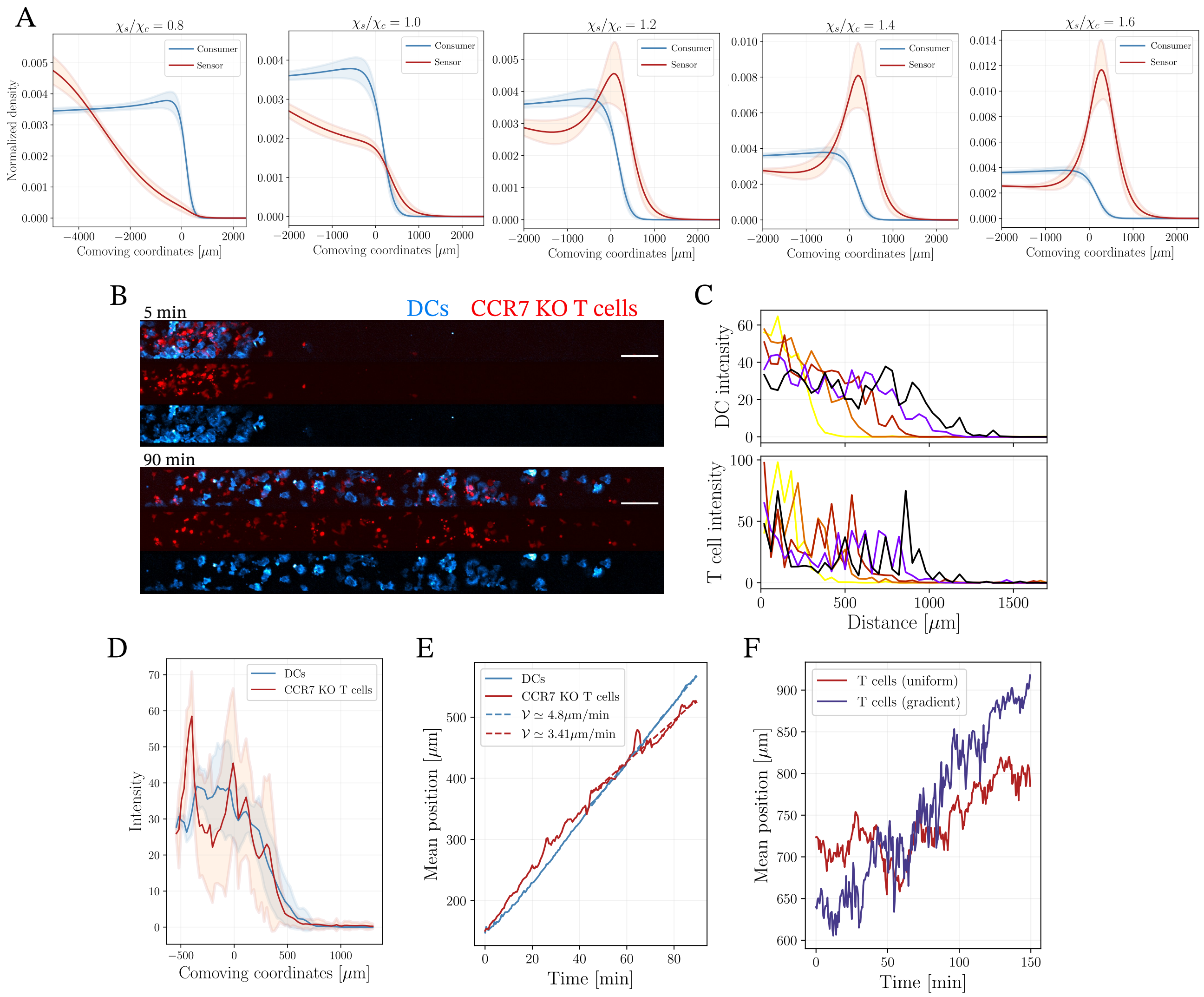

Supplementary Figure S7: **Model robustness and perturbation experiments to test coupled migration dynamics of cell populations.** A) Theoretical predictions for variations in key control parameters of the model within their estimated error ranges. Each panel shows changes in the chemotactic sensitivity ratio χ_s/χ_c, achieved by tuning χ_s for fixed χ_c within the estimated error range $\chi_s/\chi_c \simeq 1.2 \pm 0.4$ as inferred from the analysis in Fig.S6F. This analysis confirms the sharp transition to the coupled migration regime at $\chi_s/\chi_c = 1$ after which the sensor cells suddenly form a sharp density peak ahead of consumers. Estimated diffusion coefficient D_c of consumers is varied within the 95% credible intervals, together with the error obtained from the scaled diffusion coefficient of sensor cells. These parameter variations produced only minor changes in the density profiles (shaded areas show standard deviations). B) Microscopy images from the microfluidic channel experiments with labelled DCs (blue) and co-migrating CCR7-KO T cells (red) at $t = 5$min (top) and $t = 90$min (bottom). Scale bar indicates 100μm. C) Averaged intensities for DCs (top) and CCR7-KO T cells (bottom) from $n = 3$ experiments at different time points (color-coded). D) Intensity profiles at different time points overlaid in the refence frame co-moving with the mean DC position. Mean density profile of CCR7-KO T cells (red) does not exhibit a well-defined peak, while most T cell population being placed behind the propagating DC front, in contrast with the WT case shown in Fig.3 of the main text. E) Mean position of cell densities over time for DCs (blue) and CCR7-KO T cells (red) show the breakdown of the velocity coupling, where T cells now fall behind the DC population and migrate with a smaller velocity at long times. F) Mean position of WT T cell populations in the absence of a co-migrating DC population, obtained from microfluidic channel experiments with an initially uniformly distributed CCL19 field (red) and a gradient of CCL19 (purple). In both cases, the average velocity obtained from the mean position is notably smaller than that of the WT T cell populations co-migrating with DCs.

$D_s = \beta D_c$ where the scaling prefactor β was drawn from uniformly distributed values $D_s/D_c \in [2,4]$. We then varied the ratio χ_s/χ_c within the range $[0.8, 1.6]$, corresponding to the 95% percentile range of values obtained

from the experimentally inferred distribution (see Fig.S6F), together with diffusion coefficient variations. This analysis confirmed the model predictions that the coupled propagation of the density waves depended strictly on the relative chemotactic strength χ_s/χ_c, and is minimally influenced by variations in the diffusion coefficients, see Fig.S7A.

S3.4 Co-migration of dendritic cells and CCR7-KO T cells

One key prediction of the theory is that the coupled migration of the consumer and sensor cell populations is strictly controlled by their relative chemotactic coefficients, i.e. by the ratio χ_s/χ_c. More precisely, we showed that the coupled regime breaks down for $\chi_s/\chi_c < 1$ where the sensor population cannot form a well-defined peak ahead of the consumer front and falls behind. To test this prediction experimentally, we decided to use CCR7-KO T cells, which lack the receptor CCR7 that binds the chemoattractant CCL19, mixed with WT DCs to see whether their migration efficiency would be influenced by attractant-specific responses. Using microfluidic channel experiments we found that CCR7-KO T cells were not able to migrate ahead of the DCs and fell behind the DC front, even though they initially migrated ahead of DCs, see Fig.S7B-E and Supplementary Movie 2. Due to the large noise arising from a small sample size ($n = 3$ experiments) for the knock-out experiments, front position tracking based on the half-maximum of cell densities was rather amenable to large jumps so we used DC mean positions instead to define a smoother co-moving frame. Tracking the mean positions we found that sensor cell velocity was notably smaller than that of the DC front (Fig.S7E), indicating the breakdown of the velocity-coupling of traveling waves we observed in the WT experiments. Interestingly, the DCs migrated again in an approximately well-preserved density profile with a constant velocity of $\sim 4.8\mu$m/min, close to their traveling wave velocity when they migrated with WT T cells. This suggests that T cells do not act as a mechanical drag on the DC motility, and excludes such mechanical interactions between the DCs and T cells as the regulatory mechanism behind the coupled migration pattern we observed in the WT experiments.

S3.5 T cell migration in uniform and imposed gradients

Another simple prediction of the model is that, because the sensors / T cells cannot shape their own guidance signals, they should not be able to migrate as efficiently alone, i.e. in the absence of a consumer / DC population. In fact, concentration peaks of sensor populations should only exist in the mixed system with a gradient-modulating cell population. To test these predictions, we looked at the migration patterns of only T cells in microfluidic channel experiments both in uniform (equilibrated) as well as gradient setups for the chemoattractant CCL19. First, we could not observe any well-defined density peaks in either uniform or gradient setups. Second, we found that although the T cells showed quite rapid motion in a uniform CCL19 field as in the mixed setup (see Supplementary Movie 3), this was mainly due to their random, undirected movements. Indeed, their mean velocity was about 1μm/min in the uniform field (see Fig.S7F, red line), strongly deviating from their dynamics in the mixed setup where they co-migrated with DCs with velocities of 5μm/min. Interestingly, even in the pre-patterned gradient case, their mean velocity remained around 2μm/min (see Fig.S7F, purple line), suggesting that the

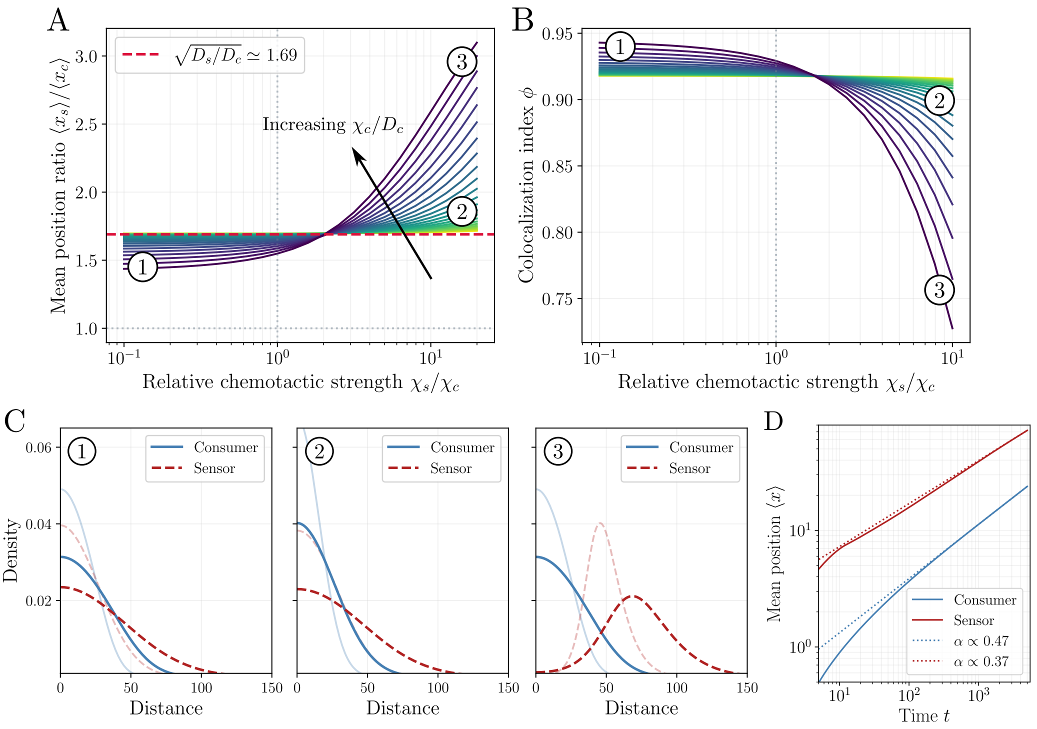

Supplementary Figure S8: **Migration and colocalization patterns in the open system with attractant turnover.** A) Mean position ratio $x \equiv= \langle x_s \rangle / \langle x_c \rangle$ as a function of relative chemotactic strength χ_s/χ_c between sensor and consumer populations for different values of chemotaxis-to-diffusion ratio of consumers (color-coded). In the uncoupled regime with $\chi_s < \chi_c$, mean position ratio is largely independent of χ_c/D_c and takes values close to the that dictated by diffusive spreading, i.e. $\bar{x} \simeq \sqrt{D_s/D_c}$. For strongly chemotactic consumers with a large χ_c/D_c, increasing the chemotactic strength of sensors (large χ_s/χ_c) leads to deviations from diffusive spreading with $\bar{x} > \sqrt{D_s/D_c}$. B) Colocalization between consumer and sensor population as quantified by the index ϕ is maximal in the uncoupled regime with $\chi_s < \chi_c$ and monotonically decays with increasing χ_s/χ_c. For strongly chemotactic consumer cells with a large χ_c/D_c, the colocalization is most sensitive to variations in the relative chemotactic strength χ_s/χ_c. C) Cell density profiles from different parameter regions plotted in (A) and (B) exhibit the emergence of pulse-like propagation for sensor cells only for large χ_c/D_c and large χ_s/χ_c. Consumer cells exhibit similar profiles for all parameter choices, and do not form pulse-like patterns. Parameter values used for the density profiles were $\tilde{\chi}_c \simeq 0.014$ and $\tilde{\chi}_s \simeq 0.28$ (Region 1); $\tilde{\chi}_c \simeq 1.4$ and $\tilde{\chi}_s \simeq 0.18$ (Region 2); $\tilde{\chi}_c \simeq 1.4$ and $\tilde{\chi}_s \simeq 22$ (Region 3). D) For strongly chemotactic consumer and sensor populations with $\chi_c/D_c \simeq 20$ and $\chi_s/\chi_c \simeq 16$, mean position of cell densities over time ($\bar{x} \propto t^{\alpha}$) shows that the long-time scaling exponents are below 0.5 and $\alpha_s < \alpha_c$.

velocity-coupling in the mixed setup provides an efficient way for the sensors to migrate over long distances.

S4 Migration patterns in the open system

In the open system in contact with an external chemoattractant reservoir, additional to its local internalization by consumer cells, the attractant evolution is regulated by a turnover rate that shifts its global level to a target concentration, see Eq.(S11). Therefore, local gradients dynamically shaped by the consumers will be directly influenced by these additional effects. To explore this, we numerically evaluated the nondimensional Eq.(S12) and analyzed the migration patterns in different parameter regions.

Phase diagram of relative positions and colocalization. We first looked at how the sensor-to-consumer mean position ratio $\bar{x} \equiv \langle x_s \rangle / \langle x_c \rangle$ was influenced in the open system: We found that in the uncoupled regime with

$\chi_s < \chi_c$, where sensor cells are less chemotactic than consumers, mean position ratio always attained values close to $\bar{x} \simeq \sqrt{D_s/D_c}$ dictated by diffusion regardless of the consumer chemotactic strength χ_c/D_c (see Fig.S8A). This is in strong contrast with the behavior we observed for the closed system, where increasing χ_c/D_c in the uncoupled regime leads to a strong decrease in $\bar{x}$, because consumer cells can form a well-defined density peak and migrate arbitrarily ahead of sensors. For large χ_s/χ_c and strongly chemotactic consumer cells with large χ_c/D_c in the open system, we observed that sensor cells now could propagate ahead of consumer in a "pulse-like" density (see Fig.S8C), leading to mean position ratios $\bar{x} > \sqrt{D_s/D_c}$ (see Fig.S8A). Furthermore, density profiles over time showed that consumer cells never formed density peaks and were spreading over time with a large concentration confined at the boundary, see Fig.S8C for the spatiotemporal profiles at different parameter regimes.

Next, we analyzed the colocalization patterns of the two cell populations by using the colocalization metric ϕ based on Jensen-Shannon divergence (see Section S5 below for details). We found that in the uncoupled regime with $\chi_s < \chi_c$ the colocalization was maximal with values $\phi > 0.9$, indicating that this regime was indeed dominated by the diffusive behavior of the two cell populations (see Fig.S8B and density profiles in Region 1 in Fig.S8C). Large values of the consumer chemotactic strength χ_c/D_c only resulted in negligible changes in ϕ. For large χ_c/D_c, increasing the relative chemotactic strength of sensors, i.e. for large χ_s/χ_c, led to a strong decrease in colocalization due to the pulse-like propagation of the sensors ahead of the consumer cells (see density profiles in Regions 2 and 3 in Fig.S8C).

To better understand the dynamics of the open system where a sensor cell pulse could be formed, we looked at the mean position of the two cell populations over time for large χ_c/D_c and large χ_s/χ_c. We found that despite the relatively large value chosen for the chemotactic strength of consumers ($\chi_c/D_c \simeq 20$), the scaling exponent for the mean position (via $\langle x_c \rangle \propto t^{\alpha_c}$) exhibited values around $\alpha_c \simeq 0.47$, indicating slightly sub-diffusive migration, see Fig.S8D. This was in strong contrast with the closed system, where $\chi_c/D_c \simeq 20$ leads to scaling exponents of $\alpha \simeq 0.7$. Furthermore, the scaling exponent of the sensor cells was around $\alpha_s \simeq 0.37 < \alpha_c$, which implied that sensors were being slowed down by the (sub-)diffusive behavior of the consumers in the back of the sensor peak.

S5 Jensen-Shannon divergence for colocalization

In addition to the co-migration efficiency of coupled cell populations, another important question is to what degree these different populations spatially overlap with each other, as this would directly influence any mechanical or contact-based signaling interactions. Particularly in the context of immune response, frequent physical contacts between DCs and T cells allow DCs to present antigens and to activate T cells [19]. To quantify the spatial overlap between cell populations, we reasoned that the colocalization metric should encode both the spatial proximity as well as the similarity in the shapes of the density profiles. For instance, the spatial overlap between a pulse-like and diffusive density profile should indicate a smaller colocalization than the overlap between two

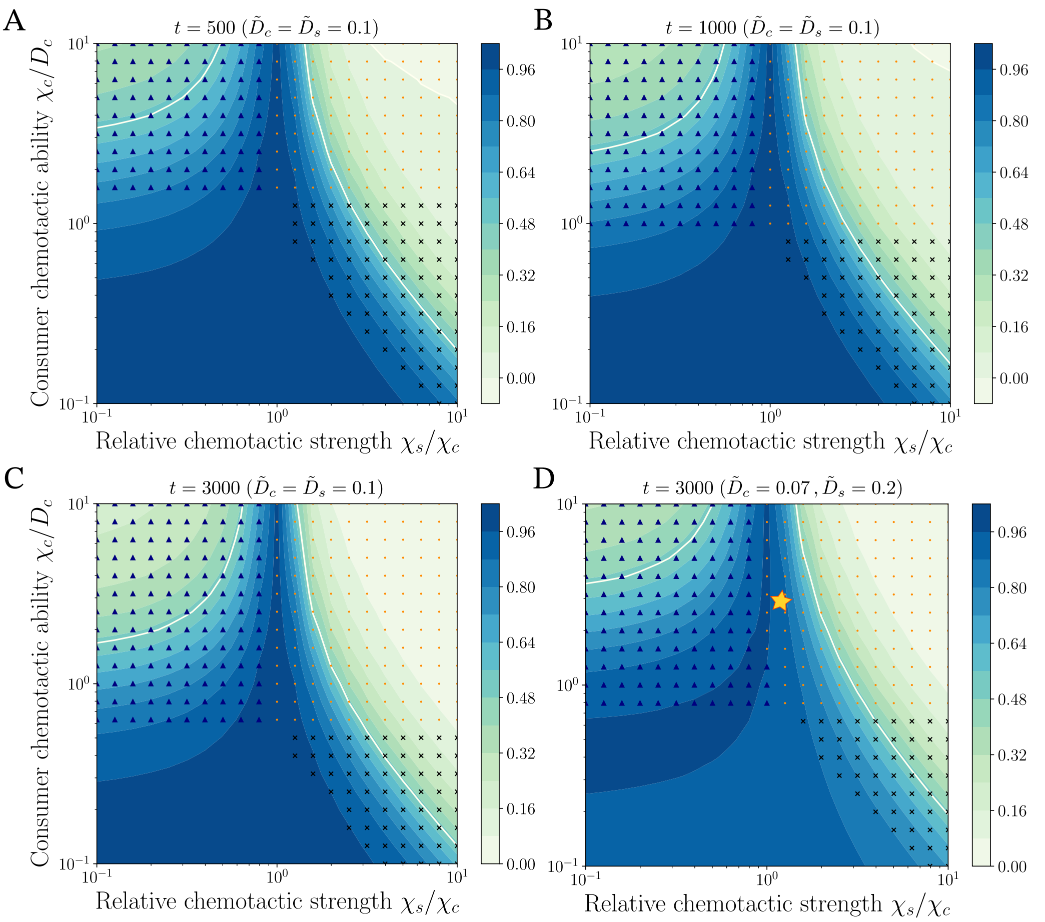

Supplementary Figure S9: **Stability of the phase diagram for the colocalization of consumer and sensor cell populations.** A-C) Colocalization index ϕ evaluated from the cell density profiles at time points $t = 500$ (A), $t = 1000$ (B), and $t = 3000$ (C) for the case of identical diffusion coefficients $\tilde{D}_c = \tilde{D}_s = 0.1$. The phase diagram remains largely preserved in particular in the coupled regime with $\chi_s/\chi_c > 1$. As t increases, density profile peaks for consumer (triangular markers) and both cell types (dots) occur in larger regions of the parameter space. D) For different diffusion coefficients as inferred from the experimental data (with $\tilde{D}_c = 0.07$ and $\tilde{D}_s = 0.2$), the colocalization is mainly influenced in the uncoupled regime of the phase space with $\chi_s/\chi_c < 1$. For weakly chemotactic consumer cells with $\chi_c/D_c < 1$ the colocalization of both cell populations is reduced in comparison with the case of identical diffusion coefficients. The parameter set for the comparison with the experimental system (star symbol) indicates a colocalization of $\phi \simeq 0.9$.

pulse-like or two diffusive profiles, as the latter two cases would lead to maximal overlap of densities. Such a metric can be defined by considering the cell concentrations as probability distributions $\rho_i(x) \to P_i(x)$ with a suitable normalization, i.e. $P_i(x) \equiv \rho_i(x)/\sum_x \rho_i(x)$, where $i = c, s$ denotes the consumer and sensor cell populations, respectively. We can then use the Jensen-Shannon divergence [20] to describe the similarity between the two probability distributions, defined by

$$D_{JS}(P_c, P_s) \equiv \frac{1}{2}\left(D_{KL}(P_c, M) + D_{KL}(P_s, M)\right), \tag{S22}$$

where $M(x) = \frac{1}{2}\left(P_c(x) + P_s(x)\right)$ and $D_{KL}(P, Q) \equiv \sum_x P(x) \log\left(\frac{P(x)}{Q(x)}\right)$ is the Kullback-Leibler divergence. Note that, we do not make use of divergence metrics for continuous random variables, as we discretize space in finite intervals of Δx to obtain the solutions of the coupled PDE system (see Section S1.1 for details on the numerical methods). Finally, as $D_{JS} = 0$ indicates maximal similarity while $D_{JS} = 1$ corresponds to zero overlap (using base 2 logarithm), we use the more intuitive choice $\phi = 1 - D_{JS}$ as a metric for colocalization.

Because the comparison of cell densities requires fixing a certain time point $t = t'$, their colocalization ϕ will in principle change dynamically as the cells migrate over space. However, as we had found for the phase diagram of relative mean positions, see Fig.S2, the phase space for colocalization ϕ also remains relatively preserved over time. Figs.S9A-C show the colocalization phase diagram evaluated at different time points for consumer and sensor cell populations with identical diffusion coefficients $\tilde{D}_c = \tilde{D}_s = 0.1$. As mentioned in the main text, using experimentally inferred values for the rescaled diffusion coefficients mainly shifts the colocalization values ϕ in the diffusive and uncoupled regime with $\chi_c/D_c < 1$ and $\chi_s/\chi_c < 1$, see Fig.S9D.

S6 Mechanical interactions between cell populations

To explore the potential role of mechanical interactions such as cell-cell adhesion or density sensing between cell populations, we modified the coarse-grained chemotaxis description to include an additional advective flux term. We chose a minimal form that allows each cell population to linearly read off the density gradient of the other, and to become either attracted or repelled by it. In the nondimensionalized form, the equations for the consumer and sensor cell density evolution then read:

$$\begin{aligned} \partial_t \rho_c &= \tilde{D}_c \nabla^2 \rho_c - \tilde{\chi}_c \nabla \cdot (\rho_c \nabla a/a) + \tilde{\mu}_c \nabla \cdot (\rho_c \nabla \rho_s)\,, \\ \partial_t \rho_s &= \tilde{D}_s \nabla^2 \rho_s - \chi_s \nabla \cdot (\rho_s \nabla a/a) + \tilde{\mu}_s \nabla \cdot (\rho_s \nabla \rho_c)\,, \end{aligned} \tag{S23}$$

where $\tilde{\mu}_c \equiv \bar{\rho}_s \mu_c / D_a$ and $\tilde{\mu}_s \equiv \bar{\rho}_c \mu_s / D_a$ are the rescaled mechanical coupling parameters with the reference consumer and sensor cell densities $\bar{\rho}_c$ and $\bar{\rho}_s$, respectively. The rescalings for t, x, D_i and χ_i can be performed analogously to the original system of equations without mechanical coupling (see Eqs.S3-S4). Note that, for clarity we will drop the tildes in the following (as is done in the main text). With this formulation, positive values ($\mu > 0$) indicate repulsion, and negative values ($\mu < 0$) indicate attraction by the other cell density. Even though this model simplifies the impact of more generic density- or contact-dependent mechanisms in collective cell migration [21], it allows for an effective screening to test the relative roles of mechanical and chemotactic interactions on the co-migration patterns. We could then numerically evaluate the system of equations Eq.(S23) together with Eq.(S4) and explore the influence of the mechanical coupling parameters μ_i on the migration patterns, see below for the details on initialization and the numerical approach.

To start testing the effect of mechanical interactions, we first set the diffusion coefficients of consumer and sensor cell types to be equal, i.e. $D_c = D_s = 0.1$, and looked at the mean position ratio $\bar{x} \equiv \langle x_s \rangle / \langle x_c \rangle$ of the two cell populations for different choices of the coupling parameters μ_i. We found that the mean position ratio $\bar{x}$ was mainly influenced by mechanical interactions for weakly chemotactic consumers (i.e. for small χ_c/D_c). Large sensor advection (large μ_s) while reducing consumer advection (small μ_c) led to a marked increase in $\bar{x}$ (Fig.S10A). This indicated that the difference between the mechanical coupling parameters $\Delta\mu \equiv \mu_s - \mu_c$ led to significant changes in the relative positions of cell populations. For strong consumer chemotaxis ($\chi_c/D_c > 1$), however, the mean position ratio $\bar{x}$ converged to predictions of the original model without mechanical coupling (Fig.S10B).

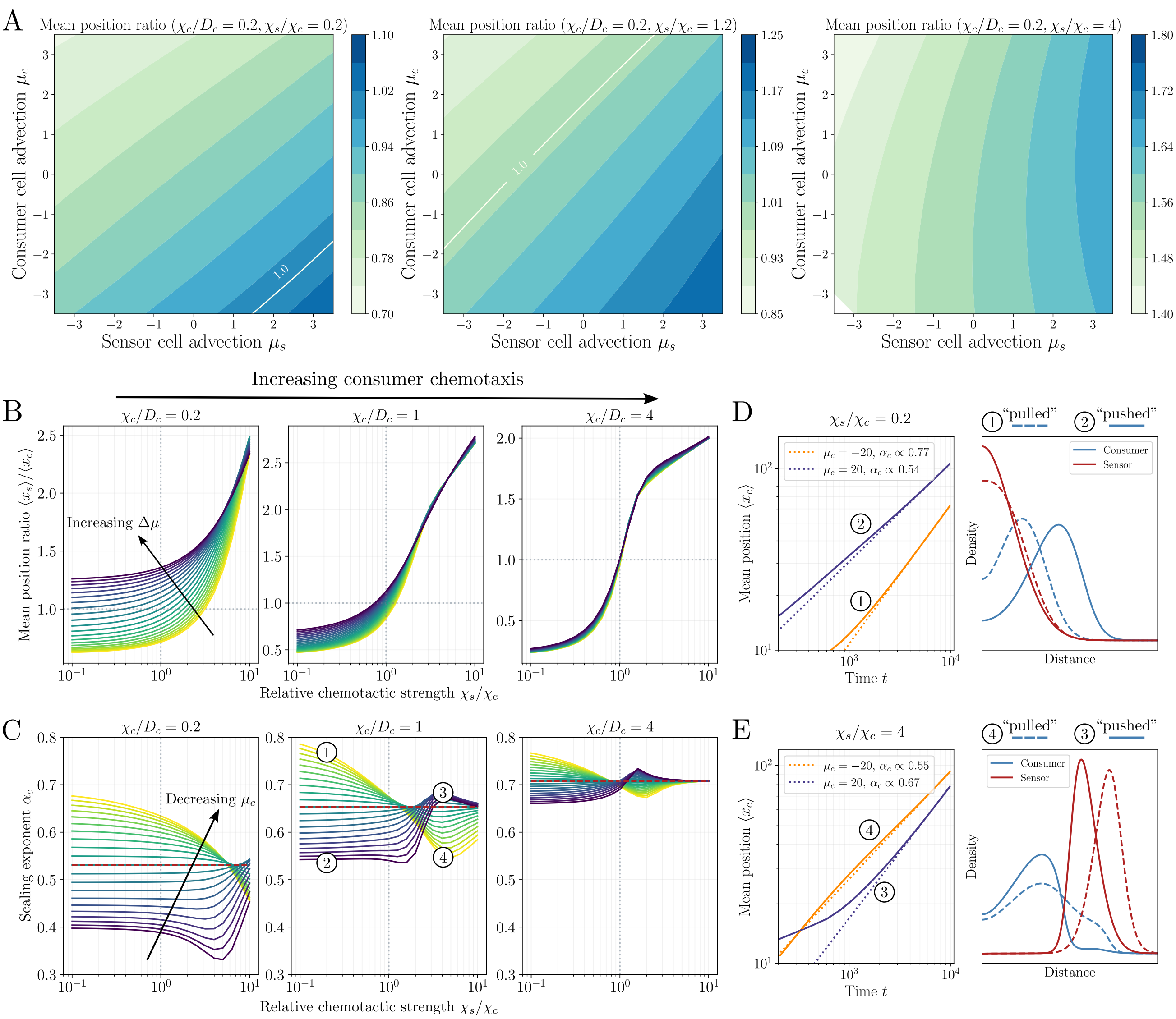

Supplementary Figure S10: **Influence of non-reciprocal mechanical interactions on the mean position ratio and long-time dynamics.** (A) Mechanical coupling, controlled by parameters μ_c and μ_s, influences the mean position ratio of cell populations in the weakly chemotactic regime of consumers ($\chi_c/D_c \simeq 0.2$). Variations in the coupling difference $\Delta\mu = \mu_s - \mu_c$ significantly affect the mean position ratio for different sensor chemotactic strengths (increasing from left to right panels). (B) For weakly chemotactic consumers (leftmost panel), large positive $\Delta\mu$ values ("pushed" sensors, "pulled" consumers) increase the relative positions. As the chemotactic ability of consumer cells increases (middle and right panel), the effect of mechanical coupling diminishes. (C) Scaling exponent α_c of the consumer cell population inferred from the long-time evolution of the mean position $\langle x_c \rangle \propto t^{\alpha_c}$ depends on the mechanical coupling parameter μ_c, with most variability observed for small χ_c and χ_s (leftmost panel). Dashed line (red) indicates the predicted values for $\mu_c = 0$. Intermediate consumer chemotaxis with $\chi_c/D_c = 1$ (middle panel) shows distinct regimes: weak sensor chemotaxis ($\chi_s/\chi_c < 1$) increases α_c with consumer attraction ($\mu_c < 0$, region 1) and decreases it with repulsion ($\mu_c > 0$, region 2). For strong sensor chemotaxis ($\chi_s/\chi_c > 1$), the trend reverses: repulsion increases α_c (region 3), while attraction decreases it (region 4). For strongly chemotactic consumers ($\chi_c/D_c > 1$, rightmost panel), mechanical coupling has a smaller effect on α_c. (D) Evolution of mean positions (left panel) and density profiles (right panel) for weakly chemotactic sensors ($\chi_s/\chi_c = 0.2$). Consumers with $\mu_c > 0$ ("pushed") exhibit more advanced mean positions than with $\mu_c < 0$ ("pulled"). Density profiles show stronger overlap between sensors and consumers for $\mu_c < 0$. (E) For strongly chemotactic sensors, consumer mean positions are more advanced for $\mu_c < 0$ than for $\mu_c > 0$ (left panel). However, for $\mu_c < 0$, consumer density profiles lose their sharp fronts (right panel).

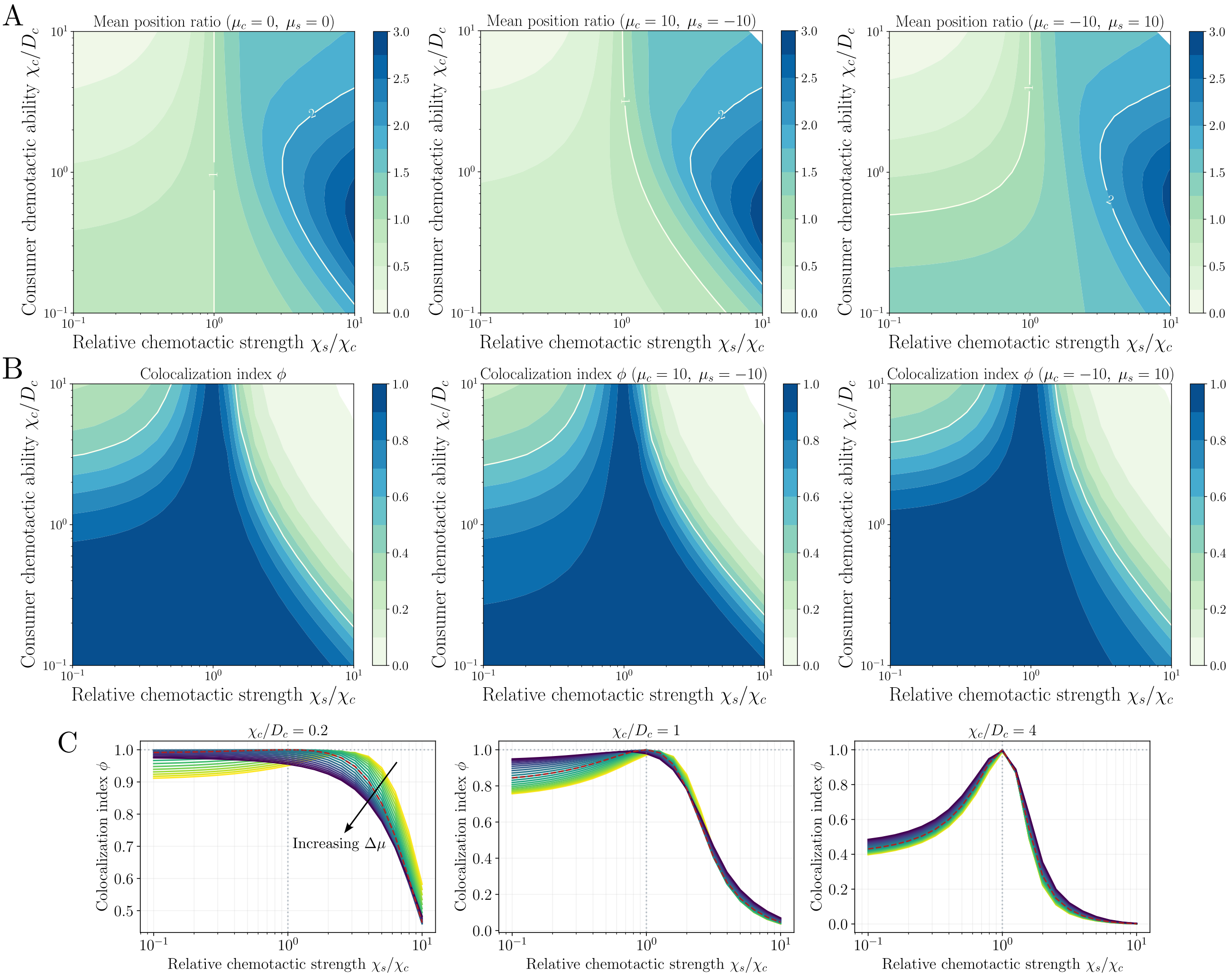

Supplementary Figure S11: **Influence of non-reciprocal mechanical interactions on the phase diagrams of mean position ratio and colocalization of cell populations.** (A) Phase diagram of mean positions changes primarily for weakly chemotactic consumer cells with $\chi_c/D_c \leq 1$ in the presence of mechanical interactions, as determined by the coupling parameter $\Delta\mu$. Contour line delineating equal mean position ratio at $\chi_s/\chi_c = 1$ without mechanical coupling ($\Delta\mu = 0$, left panel) shifts to larger χ_s/χ_c values for $\Delta\mu < 0$ (middle panel) and to smaller χ_s/χ_c values for $\Delta\mu > 0$ (right panel) in for weak consumer chemotaxis. (B) Colocalization index ϕ phase diagram shows small changes for small $\Delta\mu < 0$ (middle panel) and large $\Delta\mu > 0$ (right panel) in the weak consumer chemotaxis regime with $\chi_c/D_c \leq 1$. (C) Colocalization index for different values of the differential mechanical coupling parameter $\Delta\mu$ indicates that variations in $\Delta\mu$ only lead to small changes in the colocalization index ϕ for $\chi_c/D_c \leq 1$ (left and middle panales). Dashed line (red) represents the case $\Delta\mu = 0$. For strong consumer chemotaxis ($\chi_c/D_c = 4$, right panel) colocalization index converges to the predicted values in the absence of mechanical interactions with $\Delta\mu = 0$.

Next, we analyzed the long-time dynamics of the consumer mean position quantified by the scaling exponent α_c given by $\langle x_c \rangle \propto t^{\alpha_c}$. We found that, for intermediate consumer chemotaxis ($\chi_c/D_c = 1$), distinct migration regimes emerged depending on sensor chemotactic strength: (i) When sensor cells are weakly chemotactic ($\chi_s/\chi_c < 1$) and lag behind the consumer population, consumer attraction to sensors ($\mu_c < 0$) increased the scaling exponent α_c, while repulsion ($\mu_c > 0$) decreased it. (ii) In contrast, for strongly chemotactic sensor cells ($\chi_s/\chi_c > 1$) that migrate ahead of consumers, consumer repulsion by sensors ($\mu_c > 0$) increased α_c, while attraction ($\mu_c < 0$) decreased it (Fig.S10C-E).

To understand the influence of mechanical interactions on the original phase diagram of relative positions,

we kept $|\mu_c| = |\mu_s|$ and varied the nonreciprocity parameter $\Delta\mu \equiv \mu_s - \mu_c$. We explored limits of small $\Delta\mu < 0$ (large $\mu_c > 0$ and small $\mu_s < 0$), indicating consumers being pushed by sensors while the latter are attracted, and large $\Delta\mu > 0$ (with small $\mu_c < 0$ and large $\mu_s > 0$), which indicated consumers are pulled and sensors are pushed. We again confirmed that for both extreme choices, the mean position ratio was primarily influenced by mechanical interactions for weakly chemotactic consumer cell populations with $\chi_c/D_c < 1$, see Fig.S11A.

Finally, we asked whether the colocalization between cell populations was sensitive to variations in the mechanical interactions, as these interactions directly influence the shape of cell density profiles. Surprisingly, for the same set of of $\Delta\mu$ values as before, we found that the colocalization index was not markedly influenced by mechanical interactions, even in the weakly chemotactic regime of consumer cells, see Fig.S11B. A finer parameter scan for different values of $\Delta\mu$ while changing the strength of consumer cell chemotaxis indicated that only for small $\chi_c/D_c \leq 1$, changes in $\Delta\mu$ led to minor variations in the colocalization index ϕ, while for strong consumer chemotaxis with $\chi_c/D_c = 4$ mechanical coupling did not influence the colocalization index, which converged to values predicted in the original system without mechanical interactions, see Fig.S11C.

Numerical methods for chemotaxis with mechanical interactions. To solve the PDE system corresponding to the case including mechanical interactions (see Eqs.(S23)), we first realized that if additional advective fluxes controlled by the mechanical parameter μ were sufficiently large, this led to numerical instabilities with the finite difference scheme for initially steep density profiles as chosen before. We therefore decided to use the built-in function *NDSolve* in *Wolfram Mathematica 14.0* (Wolfram Research, Inc., Mathematica, Version 14.1, Champaign, IL (2024)). We furthermore chose smoother initial cell density profiles given by a Gaussian distribution $\rho_i(x, t = 0) = \sqrt{2\pi\sigma^2}^{-1} \exp(-\frac{1}{2}x^2/\sigma^2)$ with $\sigma = 10$. We first controlled whether this choice led to any changes in the dynamic evolution of the migration behavior in the absence of mechanical interactions and found that the phase diagrams remained largely unchanged, compare e.g. Fig.S2A with Fig.S11A (left panel), as well as Fig.4 (main text) with Fig.S11B (left panel). Having established this quantitative consistency, we then numerically solved the system of PDEs for different choices of mechanical coupling parameters μ_c and μ_s until a sufficiently large time of $T \propto 5000 - 10000$ is reached.

S7 List of Supplementary Movies

- Supplementary Movie 1 - Collective Migration of DCs and T cells
- Supplementary Movie 2 - Collective Migration of DCs and CCR7 KO T cells
- Supplementary Movie 3 - T cell migration in uniform CCL19

References

[1] Clifford S Patlak. Random walk with persistence and external bias. *The bulletin of mathematical biophysics*, 15:311–338, 1953.

[2] Thomas Hillen and Kevin J Painter. A user's guide to pde models for chemotaxis. *Journal of mathematical biology*, 58(1):183–217, 2009.

[3] Evelyn F Keller and Lee A Segel. Initiation of slime mold aggregation viewed as an instability. *Journal of theoretical biology*, 26(3):399–415, 1970.

[4] Evelyn F Keller and Lee A Segel. Traveling bands of chemotactic bacteria: a theoretical analysis. *Journal of theoretical biology*, 30(2):235–248, 1971.

[5] Casimir Emako, Charlène Gayrard, Axel Buguin, Luís Neves de Almeida, and Nicolas Vauchelet. Traveling pulses for a two-species chemotaxis model. *PLoS computational biology*, 12(4):e1004843, 2016.

[6] Xiongfei Fu, Setsu Kato, Junjiajia Long, Henry H Mattingly, Caiyun He, Dervis Can Vural, Steven W Zucker, and Thierry Emonet. Spatial self-organization resolves conflicts between individuality and collective migration. *Nature Communications*, 9(1):1–12, 2018.

[7] M Mehdi Salek, Francesco Carrara, Vicente Fernandez, Jeffrey S Guasto, and Roman Stocker. Bacterial chemotaxis in a microfluidic t-maze reveals strong phenotypic heterogeneity in chemotactic sensitivity. *Nature communications*, 10(1):1877, 2019.

[8] Henry H Mattingly and Thierry Emonet. Collective behavior and nongenetic inheritance allow bacterial populations to adapt to changing environments. *Proceedings of the National Academy of Sciences*, 119(26):e2117377119, 2022.

[9] Avaneesh V Narla, Jonas Cremer, and Terence Hwa. A traveling-wave solution for bacterial chemotaxis with growth. *Proceedings of the National Academy of Sciences*, 118(48):e2105138118, 2021.

[10] Jonas Cremer, Tomoya Honda, Ying Tang, Jerome Wong-Ng, Massimo Vergassola, and Terence Hwa. Chemotaxis as a navigation strategy to boost range expansion. *Nature*, 575(7784):658–663, 2019.

[11] Ricard Alert, Alejandro Martínez-Calvo, and Sujit S Datta. Cellular sensing governs the stability of chemotactic fronts. *Physical review letters*, 128(14):148101, 2022.

[12] Wim Van Saarloos. Front propagation into unstable states. *Physics reports*, 386(2-6):29–222, 2003.

[13] Zhi-An Wang. Wavefront of an angiogenesis model. *Discrete & Continuous Dynamical Systems-Series B*, 17(8), 2012.

[14] Jonna Alanko, Mehmet Can Uçar, Nikola Canigova, Julian Stopp, Jan Schwarz, Jack Merrin, Edouard Hannezo, and Michael Sixt. Ccr7 acts as both a sensor and a sink for ccl19 to coordinate collective leukocyte migration. *Science Immunology*, 8(87):eadc9584, 2023.

[15] J.D. Murray. *Mathematical Biology: I. An Introduction*. Interdisciplinary Applied Mathematics. Springer New York, 2007.

[16] David Selmeczi, Stephan Mosler, Peter H Hagedorn, Niels B Larsen, and Henrik Flyvbjerg. Cell motility as persistent random motion: theories from experiments. *Biophysical journal*, 89(2):912–931, 2005.

[17] Daniel Foreman-Mackey, David W Hogg, Dustin Lang, and Jonathan Goodman. emcee: the mcmc hammer. *Publications of the Astronomical Society of the Pacific*, 125(925):306, 2013.

[18] Mélina L Heuzé, Pablo Vargas, Mélanie Chabaud, Maël Le Berre, Yan-Jun Liu, Olivier Collin, Paola Solanes, Raphaël Voituriez, Matthieu Piel, and Ana-Maria Lennon-Duménil. Migration of dendritic cells: physical principles, molecular mechanisms, and functional implications. *Immunological reviews*, 256(1):240–254, 2013.

[19] Sabine Stoll, Jerome Delon, Tilmann M Brotz, and Ronald N Germain. Dynamic imaging of t cell-dendritic cell interactions in lymph nodes. *Science*, 296(5574):1873–1876, 2002.

[20] Jianhua Lin. Divergence measures based on the shannon entropy. *IEEE Transactions on Information theory*, 37(1):145–151, 1991.

[21] Kevin J Painter, JM Bloomfield, JA Sherratt, and A Gerisch. A nonlocal model for contact attraction and repulsion in heterogeneous cell populations. *Bulletin of mathematical biology*, 77:1132–1165, 2015.
